# Pathogenic modification of plants enhances long‐distance dispersal of nonpersistently transmitted viruses to new hosts

**DOI:** 10.1002/ecy.2725

**Published:** 2019-05-21

**Authors:** Ruairí Donnelly, Nik J. Cunniffe, John P. Carr, Christopher A. Gilligan

**Affiliations:** ^1^ Department of Plant Sciences University of Cambridge CB2 3EA Cambridge UK

**Keywords:** acquisition, epidemiology, inoculation, manipulation, plant virus, vector behavior

## Abstract

Aphids spread the majority of plant viruses through nonpersistent transmission (NPT), whereby virus particles attach transiently to these insects’ probing mouthparts. Virus acquisition from infected plants and inoculation to healthy host plants is favored when aphids briefly probe plant epidermal cells. It is well established that NPT virus infection can alter plant–vector interactions, and, moreover, such pathogen modifications are found in a range of plant and animal systems. In particular, viruses can make plants more attractive to aphids but inhibit aphid settling on infected plants. It is hypothesized that this viral “reprogramming” of plants promotes virus acquisition and encourages dispersal of virus‐bearing aphids to fresh hosts. In contrast, it is hypothesized that virus‐induced biochemical changes encouraging prolonged feeding on infected hosts inhibit NPT. To understand how these virus‐induced modifications affect epidemics, we developed a modeling framework accounting for important but often neglected factors, including feeding behaviors (probing or prolonged feeding) and distinct spatial scales of transmission (as conditioned by wingless or winged aphids). Analysis of our models confirmed that when viruses inhibit aphid settling on infected plants this initially promotes virus transmission. However, initially enhanced transmission is self‐limiting because it decreases vector density. Another important finding is that virus‐induced changes encouraging settling will stimulate birth of winged aphids, which promotes epidemics of NPT viruses over greater distances. Thus our results illustrate how plant virus modifications influence epidemics by altering vector distribution, density, and even vector form. Our insights are important for understanding how pathogens in general propagate through natural plant communities and crops.

## Introduction

Aphids vector the majority of arthropod‐transmitted plant viruses and about 30% of known plant viruses (Gray and Banerjee 1999, Brault et al. 2010). Some aphid‐transmitted viruses circulate within vectors and a few can infect insect cells (persistent transmission, PT; for a glossary of specialist terms used in this paper, see Box 1). However, most aphid‐transmitted viruses do not circulate internally and cannot infect the insect. Instead, virus particles attach loosely to the insect's piercing mouthparts (stylets), a form of vectoring called nonpersistent transmission (Gray and Banerjee 1999, Brault et al. 2010; NPT: Box 1). The aphid–NPT virus interaction is ephemeral, and virus particles are rapidly flushed out of the stylet channel when aphids salivate into plant epidermal cells (Powell 2005, Krenz et al. 2015). Virus acquisition is most efficient when aphids briefly sample the virus‐laden cells of an infected plant's epidermis in exploratory probes and then disperse quickly to another host, having rejected the plant as unpalatable (Powell 2005, Krenz et al. 2015). Conversely, prolonged feeding from phloem tissue of the virus is thought to hinder the spread of NPT virus (Mauck 2016, Groen et al. 2017). Hence, the epidemiology of NPT viruses, and as such the epidemiology of diverse virus species infecting a wide range of host plants, is intimately related to the behavior of their aphid vectors (initial surface probes vs. deeper feeding) as they move among potential host plants.

Box 1. Definitions of the main plant virus types together with several phenomena that we identify as key to virus epidemics.

**Table:** 

Glossary of specialist terms
NPT, nonpersistent transmission
NPT virus is carried between plants by loosely binding to the mouthparts of aphid vectors
PT, persistent transmission
PT virus circulates in and may reproduce within the vector
Aphid feeding dispersal
Departure from no‐longer‐desirable plant and re‐location, ultimately with feeding, to new host plant
VMPP, virally modified plant phenotypes
Virus‐induced effects on infected plants that alter plant–vector relations
Plant attractiveness VMPP
Virus‐infected plants attract (or repel) aphids because of changes in chemical signals emitted by plants
Plant acceptability VMPP
Aphids probing infected leaves are deterred (or retained) by effects of infection on plant palatability
Inoculum released (from host patch)
The number of winged aphids bearing NPT virus emigrating from the local host population

A growing body of work suggests that plant viruses manipulate vector behavior in order to enhance their own transmission (Mauck et al. 2016, Groen et al. 2017, Carr et al. 2018), and that beyond plant viruses, manipulation of vector host choice by pathogens (Gandon 2018) can have dramatic consequences for epidemiology. Several NPT viruses induce biochemical changes in their host plants that affect the host‐locating behavior of aphids, as well as the insects’ feeding behavior and growth after they alight on infected plants (Mauck et al. 2016, Groen et al. 2017, Carr et al. 2018). In this paper, we will refer to such virus‐induced effects on plant–vector relations as virally modified plant phenotypes (VMPPs; Box 1). Some important examples of VMPPs come from studies of cucumber mosaic virus (CMV). It was found that CMV‐infected squash plants (*Cucurbita pepo*) emit a blend of volatile organic compounds that attracts aphids (Mauck et al. 2010, Pickett et al. 2013), i.e., a VMPP conditioning *plant attractiveness* (Box 1). However, the plants contain increased levels of distasteful metabolites, which deter aphids from prolonged feeding following exploratory probes in which virus particles may have been acquired (Mauck et al. 2010), i.e., virus‐induced decrease in *plant acceptability* (Box 1). Similar results were obtained for cucumber, *Cucumis sativus* (Carmo‐Sousa et al. 2014), and it was found that CMV‐infected *Arabidopsis thaliana* plants were also distasteful to aphids (Westwood et al. 2013). A hypothesis has emerged that this particular combination of VMPPs, which we term “attract and deter” (Table 1), is a form of viral manipulation by which NPT viruses increase the likelihood of their transmission to new hosts (Mauck et al. 2016, Groen et al. 2017, Carr et al. 2018). However, it is not clear if “attract and deter” viral manipulation would lead to more severe epidemics, because, for instance, increases in virus acquisition do not always lead to higher incidence of virus‐infected plants (Sisterson 2008).

**Table 1:** Classification of virally modified plant phenotype (VMPP) combinations

Combinations of virally modified plant phenotypes (VMPPs)			
Plant attractiveness, ν			
Plant acceptability, ε	ν < 1	ν = 1	ν > 1
ε < 1	Repel and deter	Deter	Attract and deter
ε* = *1	Repel	Neutral effects of infection	Attract
ε > 1	Repel and retain	Retain	Attract and retain

*Notes*

Plant acceptability VMPP, denoted by ε, relates to the effect of virus‐infected plants on aphid feeding behavior. Plant attractiveness VMPP, denoted by ν, relates to the effect on alighting of aphids, through modification of plant volatile cues. For example, Attract and deter refers to aphids being preferentially attracted to alight on infected compared with healthy plants, and deterred from phloem feeding on infected plants once alighting with probing has occurred (ε < 1, ν > 1).

Nonpersistently transmitted viruses can also induce VMPPs that enhance the acceptability of hosts to aphids, i.e., promote the opposite extreme of *plant acceptability* VMPP rather than deter (Table 1). This type of manipulation is caused by inhibiting synthesis of distasteful substances and/or by increasing nutrient content in virus‐infected plants. Such effects, which can enhance aphid performance, were seen in CMV‐infected tobacco and in *A. thaliana* infected by turnip mosaic virus (Ziebell et al. 2011, Casteel et al. 2015). Because this form of VMPP would encourage aphids to settle for prolonged feeding on infected plants, it is thought to inhibit transmission of NPT viruses (Mauck et al. 2014). It has also been proposed that VMPPs that encourage aphid settling occur when NPT viruses and their hosts are poorly adapted to each other (Mauck et al. 2014).

To understand the impacts of VMPPs on virus epidemics, we developed a mathematical model that links virus‐induced effects on aphid–plant interactions to aphid behavior. We achieve this by explicitly modeling aphid ‘feeding dispersals’. In this context, a feeding dispersal is defined as the departure of an aphid from a no‐longer desirable plant and its relocation, ultimately with feeding, to a new host plant (Irwin et al. 2007; Box 1). Therefore, although feeding dispersal ends with phloem feeding on a new host plant, the preceding period may involve alighting on, probing of, and rejection of several plants. Distinguishing among key components of aphid behavior (i.e., aphids probe epidermal cells prior to feeding on phloem) and life history (i.e., aphid offspring develop to be either winged or wingless in response to local density) is essential to understand the epidemiological dynamics. To date, models for virus transmission ignore vector form (winged vs. wingless) and probing/feeding behavior. For instance, in a range of plant virus modeling studies (McElhany et al. 1995, Jeger et al. 1998, Sisterson 2008, Shaw et al. 2017), the central property of NPT, that acquisition occurs when aphids sample epidermal cells of host plants, is omitted because of a focus either on generic or persistently transmitted plant viruses.

In this paper we model the infection process as a by‐product of aphid probing of plants during feeding dispersals rather than as frequency‐dependent contacts between susceptible hosts and infected vectors. Because natural aphid populations comprise both winged and wingless individuals, we conclude by contrasting the effects of VMPPs on virus transmission and epidemic dynamics when the dispersing aphids are both winged and wingless. Thus, aphid behavior and disease progress are linked to aphid development, in particular to the production of nonwinged or winged forms (Braendle et al. 2006), and beyond this to the dynamics of an epidemic: from initial epidemic growth rates to final epidemic size.

## Methods

**Figure 1:**
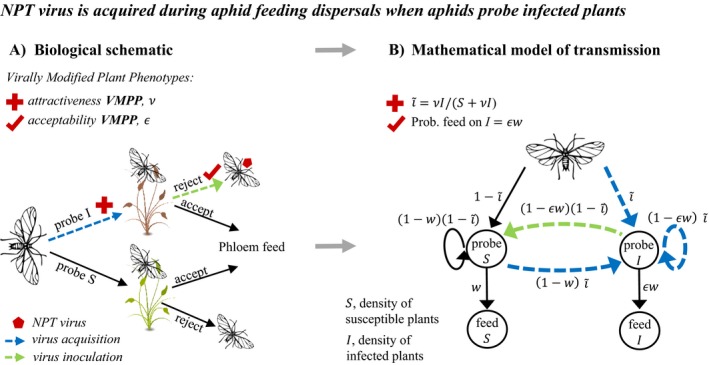
Aphid feeding dispersals where aphid feeding behavior is influenced by virally modified plant phenotypes (VMPP) of plant attractiveness (red cross) and plant acceptability (red tick). (A) Feeding dispersal is a sequence of trials involving exploratory probing of host plants by aphids where virus is potentially acquired from an infected plant and subsequently inoculated into a susceptible plant. (B) Markov chain model for virus transmission during individual feeding dispersals of aphid vectors in a population of infected (*I*) and susceptible (*S*) plants.

We first systematize the searching behavior of aphids, focusing initially on the attractiveness of plants to aphids searching for a feeding site during feeding dispersal; see glossary of specialist terms (Box 1) and biological schematic (Fig. 1A). Virus transmission (acquisition and then inoculation) is associated with alighting and probing of plant epidermal cells (Powell 2005, Krenz et al. 2015). Acquisition requires probing of an infected plant, with subsequent rejection required for NPT virus, which is only very briefly retained on the vector, to be transported to a healthy plant (blue arrow, Fig. 1A). Inoculation is associated with subsequent alighting and probing on a healthy plant (green arrow, Fig. 1A). A Markov chain for feeding dispersals of aphids (Fig. 1B) represents all possible transitions between plant probing states (i.e., probe *S* vs. probe *I*) ending in aphid acceptance of a probed plant (which corresponds to the settled state of phloem feeding). Within these representations of feeding dispersals VMPP of plant attractiveness have the effect of biasing alighting towards (or away) from infected plants and can be represented by the parameter ν (Fig. 1A, B). Plant acceptability VMPP alters the probability of acceptance of probed infected plants and can be represented by the parameter ε (Fig. 1A, B). We define a spectrum of attract–repel for plant attractiveness VMPP (ν < 1 for repel; ν > 1 for attract) and a spectrum of deter–retain for plant acceptability VMPP (ε < 1 for deter; ε > 1 for retain), see set of VMPP combinations (Table 1).

In the remainder of this section we outline the technical details required for our modeling. In outline this consists of deriving several quantities from the Markov chain in Fig. 1B. First, we show that the Markov chain leads to an expression for the expected number of transmissions per aphid dispersal and hence to an equation for disease progress (Epidemiological dynamics section). Second, we show how the Markov chain leads to expressions for the probabilities that dispersing aphids settle on different types of plants (i.e., healthy vs. virus infected) and hence to equations for vector density (*Vector dynamics* section). Together, the equations for disease progress and vector density describe the population dynamics of the pathosystem.

### Epidemiological dynamics

Analysis of the Markov chain (Fig. 1B) allows the distribution of the number of transmissions per dispersal (denoted *P*_*n*_ for *n* transmissions) to be derived (zero‐deflated geometric distribution, see Appendix S1). Taking the mean of this distribution, we find that the expected number of transmissions per dispersal is

(1) $$x\left(i\right)=\sum_{n=0}^{\infty }nP_{n}=\frac{\tilde{i}\left(1-\varepsilon w\right)\left(1-\tilde{i}\right)}{w(1-\tilde{i}\left(1-\varepsilon \right))},$$

where *w* is the probability of accepting a healthy plant after probing (i.e., entering a phloem‐feeding state) and ε*w*, accounting for deterrence (or retention) of aphids by infected plants, is the probability of accepting an infected plant after probing. In addition, *i* is the frequency of infected plants (i.e., *i* = *I*/*H*, where *I* is the density of infected plants and *H* is the total host density, which we assume is constant). Because *i* determines the probability of alighting on an infected plant in Fig. 1B, we assume that movement of the vector is mean‐field (i.e., any individual plant location in the population of host plants is visited with equal probability) for consistency with previous studies (McElhany et al. 1995, Sisterson 2008) in which winged vectors disperse to any host plant. In addition, $\tilde{i}$ is the weighted frequency, accounting for attraction (or repulsion) toward infected plants, i.e., $\tilde{i}=\nu I/\left(S+\nu I\right)=\nu i/\left(\left(1-i\right)+\nu i\right)$. Eq. (1) is key to understanding the primary benefits of VMPPs (see Appendix S2 for detailed analysis).

More generally, the Markov chains in Fig. 1B are extended to incorporate loss of aphids through emigration/mortality as well as imperfect acquisition and inoculation. We define loss of aphids through emigration/mortality as a constant probability of aphid loss per flight between plants, *p* > 0. We define imperfect acquisition and inoculation as a constant probability of virus acquisition when virus‐free aphids probe infected plants (*P*_acq_ > 0), and a constant probability of virus inoculation when virus‐bearing aphids probe healthy plants (*P*_inoc_ > 0), respectively. Equations in the main text assume *p* = 0, *P*_acq_ = 1, and *P*_inoc_ = 1 for simplicity of presentation, but the extension of these calculations to *p* ≥ 0, *P*_acq_ ≤ 1, and *P*_inoc_ ≤ 1 (Appendix S3) are used to produce the results in this paper.

For NPT viruses, the overall transmission rate is proportional to the rate of dispersing aphids per unit time, θ*A*, where θ denotes dispersal rate and *A* denotes aphid population size in a local population of host plants. Epidemics are limited by the rate at which infected plants cease being infectious (e.g., through mortality, which we assume simply leads to instant replanting with healthy plants) denoted Γ. Combining these terms, with mean transmissions from Eq. (1), the epidemic is described by an equation for the incidence of virus‐infected plants at time *t*,* i*(*t*),

(2) $$\frac{\mathrm{di}}{\mathrm{dt}}=\theta \frac{A}{H}x\left(i\right)-\Gamma i,$$

where *H* is the constant number of host plants (in Eq. (2) this converts the units of transmission into incidence of virus‐infected plants). All parameters are listed and defined in Table 2. Eq. (2) evaluated at invasion, i.e., *i* ≈ 0, leads to the following expression for the basic reproduction number for nonpersistently transmitted viruses, *R*_0_:

(3) $$R_{0}=\theta \frac{A}{H}\frac{\nu (1-\varepsilon w)}{w}\frac{1}{\Gamma },$$

which we later compare with the expression routinely taken for *R*_0_ in plant–vector–virus models (Madden et al. 2000; *Discussion* section).

**Table 2:** Summary of population variables and parameters

Definition of notation used		Units
(i) Population dynamics (plants, aphids)		
*I*	Number of infected plants	Per field
*S*	Number of healthy plants	Per field
*A*	Total aphid population size	Per field
*A*_*j*_	Aphid density	Per plant of type *j*
(ii) Aphid behavior (incorporating modification)		
ε	Settling preference (for/against probed inf. plant)	Multiplicative factor
ν	Alighting bias (toward/away inf. plant)	Multiplicative factor
*w*	Acceptance probability (healthy plant)	Prob. after probing
(iii) Additional parameters		
*H*	Total no. of hosts	Density
1/Γ	Infectious period of plants	Average time, days
*b*	Aphid mortality	Rate per day
θ	Aphid dispersal	Rate per day
*a*	Aphid reproduction	Rate per day, per capita aphid
κ	Aphid reproduction limit (upper limit on density)	Maximum aphids per plant
*p*	Aphid emigration/death as it moves between plants	Prob. per journey
*q*	Aphid survival of journey between plants, *q* = 1 − *p*	Prob. per journey
*P*_acq_	Prob. virus is acquired from infected plant	Prob. per probing visit
*P*_inoc_	Prob. virus is inoculated in susceptible plant	Prob. per probing visit

*Notes*

Mathematical and simulation models track changes in plant and aphid population variables (i). Model dynamics are influenced by VMPP combinations, field, and aphid life history parameters (ii) and (iii). The abbreviations prob. and inf. represent probability and infected, respectively.

### Vector dynamics

Viral modifications of infected plants also impact the dynamics of the vector population. In particular, viral modifications lead to relative aggregation of the vector on healthy or infected plants. This, in turn, influences the spread of the virus. Analysis of the Markov chain (Fig. 1B) leads to expressions for the probability of settling on susceptible (*S*) vs. infected (*I*) plants, denoted *F*_*S*_ and *F*_*I*_, respectively (derived in Appendix S4):

(4) $$F_{S}=S/\left(S+\nu \varepsilon I\right)=\left(1-\tilde{i}\right)/\left(1-\tilde{i}\left(1-\varepsilon \right)\right),$$

(5) $$F_{I}=\nu \varepsilon I/\left(S+\varepsilon I\right)=\varepsilon \tilde{i}/\left(1-\tilde{i}\left(1-\varepsilon \right)\right),$$

where, as per the Epidemiological dynamics section, ε and ν are distinct VMPPs conditioning plant acceptability and plant attractiveness, respectively.

Reproduction, mortality, and dispersal govern the population dynamics of phytophagous insects like aphids. Reproduction occurs when the insect is settled on the host plant with density dependence constraining insect population growth at the level of individual plants (see Hassell 1986). Combining these factors with the settling probabilities from Eqs. (4), (5), leads to equations for *A*_*S*_ and *A*_*I*_ (aphid density on the average healthy and infected plant, respectively):

(6) $$\begin{array}{c} \frac{dA_{S}}{\mathrm{dt}}=aA_{S}\left(1-A_{S}/\kappa \right)-bA_{S}-\theta A_{S}\left(1-F_{S}\right)+\theta A_{I}F_{S}i/\left(1-i\right) \end{array},$$

(7) $$\begin{array}{c} \frac{dA_{I}}{\mathrm{dt}}=aA_{I}\left(1-A_{I}/\kappa \right)-bA_{I}-\theta A_{I}\left(1-F_{I}\right)+\theta A_{S}F_{I}\left(1-i\right)/i \end{array},$$

where *a* and κ denote low‐density net growth rate and the maximum aphid density per plant for vector growth to occur. Thus the first two terms on the right‐hand side of Eqs. (6), (7) represent aphid growth (reproduction and mortality), the third term represents loss through dispersals that settle on the alternative plant type, and the fourth accounts for dispersals from the alternative plant type that settle on the focal type (note that scalings are required to divide total incoming dispersals across individual plants of a given type). Because aphid resettling, i.e., entering a feeding state on a new plant having dispersed from a different plant, occurs at the same rate as dispersal in Eqs. (6), (7), we are assuming that the plant selection process is fast compared with feeding, in both Eqs. (6), (7) and in Eq. (2). This assumption is justified by the differing orders of magnitude of feeding (h) and plant selection timescales (min; Irwin et al. 2007). Hence plant selection can be assumed to be fast relative to feeding. Total population size of the vector, at a given stage of the epidemic, *i*, satisfies

(8) $$A^{*}\left(i\right)/H=A_{S}^{*}\left(i\right)\left(1-i\left(t\right)\right)+A_{I}^{*}\left(i\right)i\left(t\right).$$

Hence we take *A* at its dynamic attractor, i.e., A*(*i*), as the epidemic, *i*(*t*), spreads. The assumption that vector density on individual plants reaches a steady‐state faster than the spread of infection among plants implies a separation of timescales for vector and infection dynamics, and that the vector is already endemic when the virus invades. Throughout, all analyses are supported with event‐based stochastic simulation, described in full in Appendix S5, which does not make any assumption of separate timescales. In addition, the assumption that the virus invades an environment where the vector is endemic is relaxed in the following section using event‐based stochastic simulation.

### Concurrent dynamics of winged and wingless aphids

A key feature of natural aphid populations is that individuals can be either winged or wingless, with winged aphids developing under crowding and with wingless the norm at low aphid density (Braendle et al. 2006). Furthermore, whereas in some situations viruses invade environments with endemic vector populations, in other situations virus and aphid invasion may occur simultaneously. To test our results with these more realistic factors we extend our event‐based stochastic simulation so that winged aphids can disperse to any host plant, but wingless aphids move only between neighboring plants. By assuming that the probability that offspring develop to be winged is a sigmoidal, increasing function of on‐plant aphid density, we allow for concurrent dynamics of winged and wingless aphids. By varying the delay between the initial immigration of a single winged aphid (which initially produces only wingless offspring at low density) and virus invasion, we contrast results from two important invasion scenarios. At one extreme, virus invasion occurs when the vector is endemic (i.e., large delay between winged aphid immigration and virus invasion), at the other extreme, vector and virus invasion coincide (i.e., no delay between winged aphid immigration and virus invasion). The spatial nature of the stochastic simulations captures spatial correlations in the incidence of virus infection. These correlations may additionally influence the impacts of VMPPs.

## Results

### What are the primary benefits of attractiveness and acceptability VMPPs?

The fundamental benefits of VMPPs conditioning plant attractiveness and plant acceptability for transmission can be seen by examining Eq. (1), mean transmissions per dispersal. Eq. (1) can be interpreted as the likelihood of acquisition and inoculation (numerator) scaled by the number of plants visited (1/denominator). Attraction toward infected plants (ν > 1) increases the numerator, and therefore increases the mean number of transmissions, for low *i*, but decreases it for high *i*. Deterrence of feeding after probing (ε < 1) increases the numerator and increases the expected number of plants visited (1/denominator), therefore increasing mean transmissions for any value of *i* (Appendix S2: Fig. S1). These patterns are the basis of suggestions that a virus inducing attraction and deterrence, i.e., attract‐and‐deter (Table 1, first row, final column, and Appendix S2) will undergo more frequent transmission than a virus that induces no change in plant phenotype (McElhany et al. 1995, Sisterson 2008).

### How do VMPPs impact epidemics, assuming constant aphid populations?

**Figure 2:**
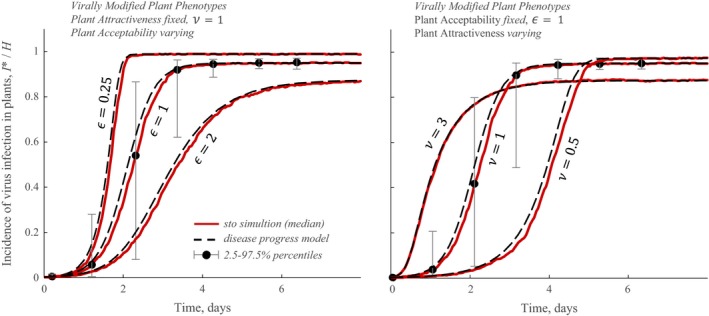
Comparison of epidemic trajectories for different VMPP combinations. (A) Trajectories are shown for contrasting values of plant acceptability VMPP, ε. Aphids have no preference between alighting on healthy and infected plants (i.e., no effect of virus infection on plant attractiveness, ν = 1). (B) Trajectories are shown for contrasting values of plant attractiveness VMPP, ν. Aphids accept healthy and infected plants with the same probability (i.e., no effect of infection on plant acceptability, ε = 1). In (A) and (B) black dashed curves represent solutions of the mathematical model (i.e., Eq. (2) was solved numerically using MATLAB version 2018a ode45 solver). Red curves represent median of event‐based simulations throughout. Therefore, both results of the mathematical model and the event‐based simulation model are presented in (A) and (B). Intervals representing the 95% percentiles are shown, for simplicity, only for ε = 1 (A) and ν = 1 (B), in both cases for several representative time points. (A) and (B) were generated with *w *=* *0.2; rates per day: θ* *= 1, Γ^−1^ = 3/20. Additionally: *P*_acq_ = *P*_inoc_ = 0.5 and *A* = 1,200. Medians were calculated from 100 simulations over 20 × 20 plants.

**Figure 3:**
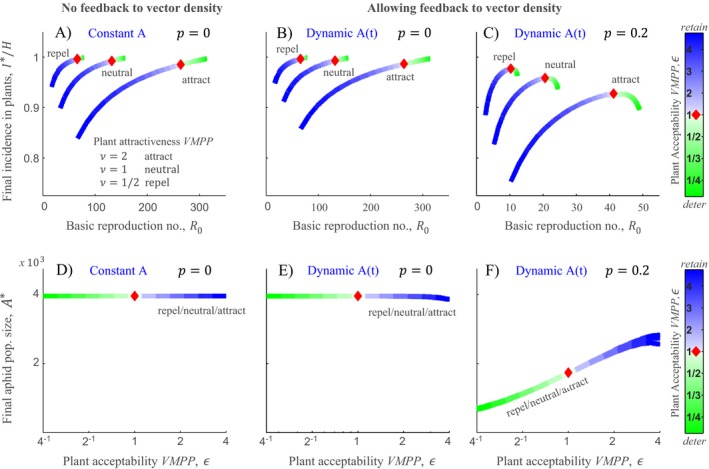
Incorporating feedback of VMPPs to vector density alters established conclusions about VMPPs. (A)–(C) show the benefits of VMPPs to NPT virus epidemics, as summarized by *R*_0_ and final incidence. (D)–(F) show the corresponding effects on vector population size. Results are contrasted across a range of assumptions relating to population dynamics of the vector. (A, D) Assuming a constant background population of aphids as is consistent with previous studies. (B, E Assuming an equilibrium background population of aphids (when virus invades) with no risk of aphid loss associated with movement between plants (*p* = 0). (C, F) Assuming an equilibrium background population of aphids (when virus invades) where there is risk of aphid loss associated with movement between plants (*p* = 0.2). In (A)–(F) there are three curves for plant attractiveness VMPP representing attract, no modification, and repel. Each curve is composed of varying plant acceptability VMPP from ε = 1/4 to ε = 4; see color bar. In (A)–(C) *R*_0_ (*x*‐axis) is calculated from Eq. (3), main text. In (A) final incidence (*y*‐axis) is calculated by setting Eq. (2) equal to 0 and solving for *i*. In (B)–(C) final incidence (*y*‐axis) is calculated by simultaneously solving Eq. (2) = 0 together with 6 and 7 equal to 0 and solving for *i* and *A*. (A)–(F) were therefore generated only from the mathematical model (Eqs. (2), (6), (7)). Parameters in (A)–(F) were: *w *=* *0.2; rates per day: Γ = 3/20, θ = 2, *b* = 1/50, *a *=* *2. Additionally *P*_acq_ = *P*_inoc_ = 0.5 and κ = 10.

When epidemiological dynamics are taken into account the epidemic is described by an equation for the incidence of virus‐infected plants, Eq. (2). Assuming that *A* is constant makes the analysis comparable to previous studies (McElhany et al. 1995, Sisterson 2008). In Fig. 2 full disease progress curves are shown, and compared with epidemic trajectories from event‐based stochastic simulations, for several representative values of plant acceptability, ε (Fig. 2A), and several representative values of plant attractiveness, ν (Fig. 2B). This illustrates the effect of these VMPPs, and confirms the accuracy of the deterministic solution (dashed curves from the mathematical solution vs. red curve for median stochastic trajectory). The epidemic is additionally summarized in Fig. 3A in terms of initial epidemic growth rate, *R*_0_ (*x*‐axis, calculated according to Eq. (3)) and final incidence (*y*‐axis; solution of Eq. (2) = 0) for a range of VMPP combinations (separate curves for values of plant attractiveness, ν, with a range of plant acceptability, ε, values within each curve). For instance, comparison of disease progress for a no‐VMPP virus and an attract and deter virus verifies that increased transmission (cf. Eq. (1)) is reflected in rapid early epidemic growth and higher final incidence.

### What are the effects of VMPPs on aphid population size?

Because in nature aphid populations are dynamic (i.e., not constant but continually changing; Eq. (2) with *A* = *A*(*t*)), a key question is how VMPPs influence aphid density. Analysis of aphid population size, once the epidemic has spread, i.e., solving Eq. (6), (7) with steady‐state *i* (itself the solution of Eq. (2) = 0), shows that overall population size is highly sensitive to retention (ε > 1) vs. deterrence (ε < 1) if *p* > 0 (Fig. 3E, F, cf. Fig. 3D). This sensitivity is seen when *p* > 0 but not when *p* = 0. When *p* = 0 (no risk of aphid loss per flight) the aphid population size is approximately constant irrespective of the level of plant acceptability VMPP, ε, and matches the value of *A* from the constant aphid background case (Fig. 3E, cf. Fig. 3D). However, the scenario of *p* = 0 is highly unrealistic, as it implies 100% escape from risks associated with movement such as rain, or exposure to wind, which can lead to dramatic displacement (Irwin et al. 2007). Therefore, too much deterrence depletes the vector population size. This novel finding emphasizes that VMPPs impact vector density, which drives our results. In contrast, we find that attraction (ν > 1) vs. repulsion (ν < 1) does not significantly alter final aphid population size.

### How do VMPPs impact epidemics assuming a dynamic aphid population?

Assuming *A* varies at a dynamic equilibrium, instead of being a constant background population size, goes beyond existing studies as a setting for studying NPT virus epidemics (i.e., we explicitly model aphid population dynamics). This case is analyzed by solving Eq. (2) with steady‐state A*(*i*) (itself the solution of Eqs. (6), (7) = 0). Crucially, if *p* = 0 the results are identical to when the aphid population size is assumed to be constant (compare Fig. 3B with A). However, when *p* > 0, the effects of VMPPs on epidemics are driven by their impacts on vector population size (cf. Fig. 3C, F), and the result is that too much deterrence reduces final incidence (Fig. 3C). Taken together, these results indicate a major cost to deterrence strategies. Although they increase the likelihood that aphids will acquire an NPT virus during superficial probes, too much deterrence is ultimately self‐limiting for the spread of infection because it depletes vector population size.

### How do VMPPs impact epidemics driven by concurrent winged and wingless aphids?

**Figure 4:**
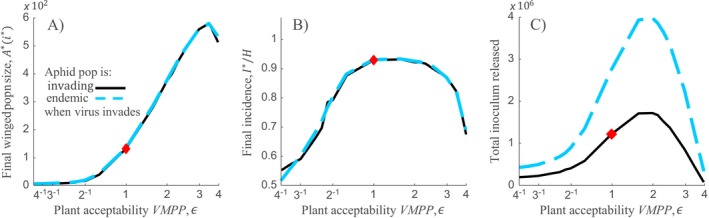
Concurrent dynamics of winged and wingless aphids, summarizing the effects of VMPPs on NPT virus epidemics when aphid and virus invasions coincide. (A) Final population size of winged aphids. (B) Final incidence of virus‐infected plants. (C) Incorporating winged and wingless aphids introduces a previously unconsidered effect of VMPP: inoculum release from the local host population. Inoculum released is the total number of virus‐bearing winged aphids that emigrated from the host patch during a 60 day NPT virus epidemic. In (A)–(C) results are shown for varying plant acceptability VMPP only (for simplicity, there was no effect of virus infection on plant attractiveness, ν = 1). Blue dashed curve in (A)–(C) shows results when vector is already endemic for comparison (i.e., at steady‐state when the virus invades). Parameters for (A)–(C) were as per caption of Fig. 3 but with *p *=* *0.2 throughout. In addition, the probability that individual offspring of aphids on any plant *i*,* j* develop to be winged was $A_{i,j}^{6}/\left(A_{i,j}^{6}+{\left(0.5\kappa \right)}^{6}\right)$. This form was chosen so that the probability that offspring develop to be winged is exactly 0.5 when aphid density on the plant (*A*_*i*,*j*_) is κ/2, and in addition, is higher than 0.95 when aphid density on the plant is κ. All figures were generated using event‐based simulation only. Results represent the median of 4,000 replicate simulations commencing with virus invasion and simultaneous introduction of a single settled winged aphid into a field of 20 × 20 otherwise uncolonized susceptible host plants. For blue dashed curves, results represent the median of 1,000 replicate simulations with virus invasion lagged so that the vector had reached steadystate at the time of virus invasion.

In reality, the population dynamics of winged and wingless aphids are concurrent: crowding stimulates production of the winged form (Braendle et al. 2006, Irwin et al. 2007). When populations are composed of winged and wingless individuals, we use simulations to extend our key findings. The distinction in aphid form in mixed populations allows us to introduce a new epidemiological measure: the level of inoculum released by the local population of host plants, i.e., number of virus‐bearing winged aphids that emigrate away from the local host population (Fig. 4C; glossary of specialist terms in Box 1). By leading to higher aphid density on infected plants, retention is associated with greater production of winged aphids (Fig. 4A). By leading to lower aphid density on infected plants, deterrence is associated with lower production of winged aphids. The incidence of virus‐infected plants and the likelihood of acquiring the virus during probes combines together with the population size of winged aphids to determine the amount of inoculum released (Fig. 4C). Therefore, the cumulative number of winged aphids bearing NPT virus emigrating over the season (Fig. 4C) is maximal for intermediate levels of aphid retention on infected plants. The blue dashed line represents, as a baseline, the case where the vector population is already endemic when the virus invades. It confirms the robustness of our results to assumptions regarding vector endemicity (Fig. 4).

## Discussion

Ideas that NPT viruses modify plant phenotypes to increase virus transmission have become increasingly influential in discussions of virus–plant–aphid interactions (Mauck et al. 2010, 2012, 2016, Carmo‐Sousa et al. 2014, Groen et al. 2017, Carr et al. 2018). Although VMPPs influence aphid behavior under experimental conditions, these phenomena have been largely ignored in epidemiological analyses (cf. Cunniffe et al. 2015, challenge 8) despite a few exceptions (see, e.g., McElhany et al. 1995, Daugherty et al. 2017, Shaw et al. 2017). However, it is not yet certain that VMPPs are genuine manipulative adaptations (sometimes referred to anthropomorphically as viral strategies) or incidental by‐products of infection (Mauck et al. 2014, 2016, Groen et al. 2017, Carr et al. 2018). On the assumption that VMPPs can arise as the result of host virus coadaptation it has been suggested that if a VMPP conditions settling of aphids on an infected plant, this inhibits NPT, and indicates that the virus and host are poorly adapted to each other (Mauck et al. 2014, 2016). In contrast, because deterrence against settling is thought to enhance NPT, it has been conjectured that when viruses engender VMPPs conditioning ‘deter’ or ‘attract and deter’ (see next section), this means that a host and virus are coadapted (Mauck et al. 2014, 2016). Our analysis does support the idea that VMPPs that first attract and subsequently deter aphids from infected plants initially increase transmission (Mauck et al. 2010, Westwood et al. 2013, Carmo‐Sousa et al. 2014). However, our analyses predict that the enhancement of initial transmission would be self‐limiting. Perhaps most importantly, we found that VMPPs that promote aphid settling, and hence reproduction (which requires aphids to be settled on host plants), lead to increased development of winged aphids, and do not inhibit NPT but, rather, are more likely to cause longer‐range virus transmission and trigger larger‐scale epidemics.

### Attract and deter initially promotes transmission but is ultimately self‐limiting

What we term attract and deter (Table 1) is a combination of VMPPs causing infected plants to become more attractive to aphids but inhospitable to settling. This VMPP combination has been seen in several plant–virus interactions and has been proposed to be a manipulative adaptation that viruses utilize to enhance their own transmission by aphids (Mauck et al. 2010, Westwood et al. 2013, Carmo‐Sousa et al. 2014). However, there are examples where NPT viruses induce a hospitable state in the host and where the performance (growth, reproduction) of aphids placed on virus‐infected plants is enhanced (Boquel et al. 2011, Ziebell et al. 2011, Casteel et al. 2015, Tungadi et al. 2017).

We have established a modeling framework that distinguishes aphid probing of epidermal cells (which promotes viral acquisition and inoculation) from prolonged phloem feeding (which prevents viral acquisition and inoculation). When we assumed a constant population size of aphids that can move anywhere in the population of plants to most closely match previous studies (McElhany et al. 1995, Sisterson 2008), our results support previous suggestions (Mauck et al. 2010, Westwood et al. 2013, Carmo‐Sousa et al. 2014) that attract and deter enhances virus transmission. However, if we instead allowed for the aphid population size to be dynamic, previous published conclusions are only supported under certain restricted conditions. Specifically, if *p* = 0, i.e., 100% of aphid movement between plants escapes emigration or dispersal mortality. But *p* = 0 is a highly unrealistic assumption, as aphids are exposed to risks associated with movement, including increased mortality and long‐distance dispersal (Irwin et al. 2007). If *p* > 0, VMPPs impact aphid population dynamics as the virus spreads among plants and previous conclusions are no longer supported. Thus, an important and novel finding is that induction by a virus of deterrence to settling is ultimately self‐limiting under this feedback, because it leads to a decrease in the vector population size.

In this work and in several previous studies (McElhany et al. 1995, Sisterson 2008) preferential attraction of aphids to virus‐infected plants increases the rate of spread of infection early in an epidemic. Later in the epidemic, attraction leads to bottlenecks as virus‐bearing aphids rarely alight on healthy plants, which leads to a lower final disease incidence (summarized in Figs. 2, 3; Appendix S2: Fig. S1, Appendix S7: Fig. S1). Like McElhany et al. (1995) and Sisterson (2008), we find that deterrence against vector feeding (an extreme of plant acceptability VMPP) increases the probability of vectors acquiring the virus when they probe infected plants. But we report an additional benefit of deterrence, which is that late in epidemics, a higher occurrence of rejection of probed plants leads to more sustained feeding dispersals. In another study, Madden et al. (2000) modeled frequency‐dependent contacts between virus‐bearing vectors and healthy plants. It was concluded by Madden et al. (2000) that for NPT viruses, *R*_0_ = ϕ^2^(*A*/*H*)(1/*Γ*)(1/*τ*), where 1/τ is the infectious period of the vector and ϕ represents plants visited per day by an aphid (note that we have set several ancillary parameters to 1 to simplify the expression). Thus, conventional models that do not account for aphid behavior (feeding vs. probing), lead to expressions for *R*_0_ for NPT viruses, as for PT viruses, that are proportional to the infectious period of both the host plant and vector, and to the square of the dispersal rate. In contrast, our framework shows that *R*_0_ should be proportional to only the plant's infectious period and that it should scale linearly with dispersal rate (Eq. (3)), because, crucially, transmission occurs within individual feeding dispersals as a by‐product of aphids probing infected plants.

These results are based on the assumption of a constant background density of aphids. But it is overly simplistic to assume that aphid population size is constant over time. Moreover, changes in aphid behavior because of VMPPs must impact aphid population size in some way. Therefore, in addition to disease progress, we modeled aphid population dynamics and, to the best of our knowledge, the results that emerged are entirely novel. We found that plant acceptability VMPPs influence vector density when movement between plants is not completely free from the risks of dispersal or mortality (Fig. 3F, *p* > 0). The consequences of this for conventional interpretations is seen in Fig. 3: there is no change to VMPP impacts when virus invades steady‐state aphid populations if *p* = 0, compared with when aphid density is constant (compare Fig. 3B with A). However, VMPP impacts are changed substantially when *p* > 0 (compare Fig. 3C with A). In particular, for the first time we show that deterrence comes at the cost of lower aphid density and that for this reason enhanced spread of the virus because of deterrence is ultimately self‐limiting.

### The epidemiological importance of the aphid transition from wingless to winged

The other extreme of plant acceptability VMPPs is retain (Table 1), which has the opposite effect to attract and deter by encouraging aphid population growth through settling. Retention is likely to foster aphid reproduction and survival on virus‐infected plants, but it is thought not likely to enhance NPT, based on current ideas (Mauck et al. 2010, Westwood et al. 2013, Carmo‐Sousa et al. 2014). Importantly, however, a retain VMPP is likely to encourage increased birth of winged aphids. In nature, wingless is the norm when density is low, but local aphid crowding leads to increased tactile contact between individuals, stimulating birth of winged aphids (Braendle et al. 2006, Irwin et al. 2007). Hence, we extended our framework to distinguish between local transmission (facilitated by wingless aphids) and longer‐distance virus transmission (facilitated by winged aphids). We found that the transient population dynamics of aphids are characterized by rapid increase in numbers of wingless aphids followed by an increase in winged aphids (Appendix S8: Fig. S1). The changing balance between winged and wingless aphid densities is critical for virus epidemics for two reasons: (1) it results in distinct movement scales, and (2) in combination with disease progress and the incidence of virus infection in plants, it determines the rate of emigration of virus‐bearing winged aphids. An important new finding is that aphid population size, and the proportion of aphids that are winged, are highly sensitive to VMPPs affecting plant acceptability. Therefore, inoculum release (i.e., export of virus bearing aphids to different fields) is also sensitive to VMPPs affecting plant acceptability. In particular, when virus‐infected plants deter feeding, substantially less inoculum is released from local host plant populations than when viruses have no effect on plant phenotypes (Fig. 4C). In addition, inoculum released is maximal for intermediate values of a retention strategy. This introduces a major new dimension to the costs of deterrence at a previously unstudied scale and, conversely, introduces an important benefit for transmission arising from VMPPs causing retention. Indirect support for this comes from experiments showing that higher aphid (*Aphis gossypii*) densities with relatively higher production of the winged form occurred on zucchini plants infected with zucchini yellow mosaic virus (an aphid transmitted NPT plant virus) than on uninoculated plants (Blua and Perring 1992).

### Potential practical applications

A potential practical use for the insights gained from the model introduced in this paper is to devise new ways of decreasing crop losses by disrupting NPT. Current aphid control methods such as insecticide application are limited in their ability to prevent NPT. In large part this is because inoculation occurs very rapidly for nonpersistently transmitted viruses and before an insecticide can take effect on the vector (Hull 2014). Some insecticides, notably the neonicotinoids, have been successfully used to inhibit aphid‐mediated virus transmission, particularly of PT viruses. The long‐term utility of such chemicals is curtailed by restricted use because of environmental concerns over toxicity (Godfray et al. 2014). There is also a high risk of evolution of insecticide resistance in aphid populations (Westwood and Stevens 2010). Understanding how virus infection itself affects transmission dynamics and what the optimal conditions are for an epidemic provides important insights to inform urgently needed new methods of impeding NPT.

It is possible to adapt our modeling approach to determine how crop plants, with different degrees of attractiveness to vectors, could be arranged so as to disrupt transmission dynamics. For example, encouraging virus‐bearing aphids to alight preferentially on decoy plants or virus‐resistant hosts would be expected to reduce the transmission of NPT virus. Accordingly, in an “attract‐to‐contain” intercropping system (see Appendix S9: Fig. S1), an agronomically desirable susceptible variety of the host plant would be intercropped with a variety that attracts aphids and carries major gene resistance to the NPT virus. Modeling could contribute to understanding the effectiveness of such systems, and to refining their deployment. For example, the proportion of attractive, resistant plants required to have a significant effect in reducing NPT epidemic spread can be estimated using the framework introduced in this paper (Appendix S9: Fig. S1). These ideas for inhibiting aphid‐mediated virus transmission borrow heavily from the highly successful “push‐pull” approach in which intercropping with insect‐attracting decoy plants has been used to inhibit spread of lepidopteran pests (Khan and Pickett 2004, Pickett and Khan 2016).

## Conclusions

We analyzed in detail the effects on NPT of the searching behavior of aphids during feeding dispersals, the role of vector dynamics (including the crowding‐induced developmental transition of wingless to winged aphids), and the influence of VMPPs. The analyses support an established hypothesis that NPT is accelerated if virus infection alters a host so that it becomes more attractive to aphids, while also stimulating it to accumulate compounds that deter aphid settling (the attract and deter VMPP; Carmo‐Sousa et al. 2014, Groen et al. 2017, Mauck et al. 2010, 2012, 2016). Importantly, however, VMPPs that deter settling put aphids at risk of being lost to the local host population (e.g., wind‐borne long‐distance movement, failure to locate another host, mortality associated with rain and wind) and do not encourage feeding and reproduction, for which settling on a host plant is required. Thus, virus transmission stimulated by attract and deter VMPPs leads to decreased aphid density and is ultimately self‐limiting. The second, potentially highly significant, outcome of the analysis refutes previous conjectures that retentive VMPPs (i.e., those that foster aphid settling) inhibit NPT. Rather, a retain VMPP may be highly efficient in launching epidemics through longer‐range virus transmission by virus‐bearing winged aphids. If attract and deter and retain are indeed the results of authentic viral manipulative adaptations or strategies, we think it plausible that both have roles to play in enhancing NPT virus transmission and epidemic development, but at distinct spatial scales.

## Data Availability

Supporting computer code is available on GitHub at https://doi.org/10.5281/zenodo.2529518.
