# Supplementary material for: Pathogenic modification of plants enhances long‐distance dispersal of nonpersistently transmitted viruses to new hosts
Source: Ecology. 2019 May 21;100(7):e02725. doi: 10.1002/ecy.2725 (PMC6619343; doi:10.1002/ecy.2725)
Supplement: Supplementary file 1 [file ECY-100-na-s001.pdf]

Appendix S1, Transmission distribution: number of transmissions per feeding dispersal.

1 Transmission distribution: derivation

In what follows we use several probabilistic conditionings in order to derive the distribution for the number of transmission events per aphid feeding dispersal from the Markov-chain in Fig 1B, main text. For simplicity of presentation, several factors are omitted here but are incorporated in Appendix S3. These factors are imperfect acquisition and inoculation probabilities, and the possibility of aphid loss per journey between plants. In the former, when an aphid probes an infected plant there is a probability of acquisition, $0 \leq P_{acq} \leq 1$ and when a virus-bearing aphid probes a healthy plant there is a probability of inoculation, $0 \leq P_{inoc} \leq 1$. When an aphid journeys between two plants there is a probability, $0 \leq p \leq 1$, that the aphid is lost from the local population. The remainder of this section assumes that $p = 0$ and that $P_{acq} = P_{inoc} = 1$ but the incorporation of these factors is shown in detail in Appendix S3. Note that the results in the main text are generated using the general cases of these factors i.e., $0 \leq P_{acq} \leq 1, 0 \leq P_{inoc} \leq 1$ and $0 \leq p \leq 1$, see figure captions, main text, for exact values of these parameters.

Our calculations involve probabilistic conditioning based on two alternative outcomes during feeding dispersal: a) inoculation b) feeding, see Fig S.1 for more details. The probability of feeding before inoculating for departure from a plant of type S is denoted by P_F^S (P_F^I from an I plant). Note that for all calculations the vector is assumed to have just probed, with the decision to feed or depart being imminent. The probability of inoculating before feeding starting from a susceptible plant is $P_k^S = 1 - P_F^S$ and starting from an infected plant is $P_k^I = 1 - P_F^I$. The pmf (probability mass function) for the number of inoculations, n, per feeding dispersal is:

$$P_0 = \tilde{s}P_F^S + \tilde{i}P_F^I \tag{S1}$$

$$P_n = (\tilde{s}P_k^S + \tilde{i}P_k^I)(P_k^S)^{n-1}P_F^S \tag{S2}$$

where $\tilde{s}$ represents the probability of the vector landing on a susceptible plant and $\tilde{i}$ on an infected plant, i.e. $\tilde{s} = S/(S + \nu I)$ and $\tilde{i} = \nu I/(S + \nu I)$, since the landing probabilities incorporate a bias, $\nu > 1$, due to *VMPP* (see Fig.1 main text for definition). We can understand the distribution given by Eq.s S1 and S2 in the following way. Since $P_{n+1} = P_k^S P_n$ and each P_n have a $P_F^S = 1 - P_k^S$ factor, Eq.s S1-S2 have the general form of a geometric distribution. However, because of P_0 and the coefficient term $(\tilde{s}P_k^S + \tilde{i}P_k^I)$ in S2, it is not balanced i.e. it may be a zero-inflated/deflated geometric (see section 2 of this appendix for proof).

A vector embarks on feeding dispersal. With probability $\tilde{s}$ it alights on an uninfected plant and probes it, alternatively with probability $\tilde{i}$ it alights on an infected plant and probes it. The probability of exactly zero inoculations in a feeding dispersal, P_0, is represented by $\tilde{s}P_F^S + \tilde{i}P_F^I$ (i.e. feeding prior to inoculating). On the other hand, if any inoculations are to occur the vector must inoculate before feeding which occurs with probability $\tilde{s}P_k^S + \tilde{i}P_k^I$ (i.e. at least one inoculation). Following the first inoculation, the vector is on an uninfected plant having just probed it (though plant will become infected). Therefore, for only one inoculation to occur the vector must next feed before inoculating, given it has just probed an uninfected plant (multiply previous probability by P_F^S), and this corresponds to Eq. S2 with $n = 1$. Alternatively, for two inoculations, following the first, the vector must inoculate before feeding, and after the second it must feed before inoculating (multiply previous probability by P_k^S and again by P_F^S). This corresponds to Eq. S2 with $n = 2$ and the same rationale applies for $n > 2$.

The inoculating and feeding probabilities P_F^S, P_F^I, P_k^S and P_k^I are calculated by conditioning on possible events:

$$P_F^S = 1.w + (1-w)\tilde{s}P_F^S + (1-w)\tilde{i}P_F^I \tag{S3}$$

$$P_F^I = 1.\epsilon w + (1-\epsilon w)\tilde{s}.0 + (1-\epsilon w)\tilde{i}P_F^I \tag{S4}$$

$$P_k^S = 0.w + (1-w)\tilde{s}P_k^S + (1-w)\tilde{i}P_k^I \tag{S5}$$

$$P_k^I = 0.\epsilon w + (1-\epsilon w)\tilde{s}.1 + (1-\epsilon w)\tilde{i}P_k^I \tag{S6}$$

For example, in Eq. S3 P_F^S is equal to the sum of three events weighted by their own probability of achieving feeding before inoculating. The events are: 1) the vector decides to feed (probability w) which is multiplied by 1 since feeding before inoculating has occurred; 2) the vector decides not to feed (probability $1-w$), and goes to a new plant which is susceptible (with probability $\tilde{s}$), multiplied by P_F^S as the vector is again in the same position as it started; 3) the vector decides not to feed (with probability $1-w$) but this time goes to an infected plant (with probability $\tilde{i}$) multiplied by P_F^I as this is the position of the vector on an I plant. Similarly for Eq.s S4-S6, however, note that the second term in the right hand side of Eq. S4 representing a vector having probed and then rejected an infected plant and having then gone to a susceptible plant and probed it, i.e. an inoculation, is weighted by 0 as this event is the opposite to feeding before inoculating.

Solving these simultaneous equations results in the following expressions

$$P_F^S = \frac{w\tilde{s} + \epsilon w\tilde{i}}{(1-(1-\epsilon w)\tilde{i})(1-(1-w)\tilde{s})} \tag{S7}$$

$$P_k^S = \frac{(1-w)\tilde{i}(1-\epsilon w)\tilde{s}}{(1-(1-\epsilon w)\tilde{i})(1-(1-w)\tilde{s})} \tag{S8}$$

$$P_F^I = \frac{\epsilon w}{1-(1-\epsilon w)\tilde{i}} \tag{S9}$$

$$P_k^I = \frac{(1-\epsilon w)\tilde{s}}{1-(1-\epsilon w)\tilde{i}} \tag{S10}$$

Eq.s S7-S10 together with S1-S2 give a closed form for the distribution for the number of inoculations per feeding dispersal under an assumption of mean-field vector movement.

2 NPT transmission distribution: zero-inflated vs zero-deflated geometric distribution

If the transmission distribution were balanced (i.e. neither inflated nor deflated) then the $n = 0$ and $n = 1$ terms of the distribution would be related as $P_0 = P_1/P_k^S$. However, according to the distribution represented by Eq.s S1 - S2

$$\begin{aligned} P_1 &= (1 - P_0)P_F^S \\ \Leftrightarrow P_0 &= 1 - P_1/P_F^S \end{aligned} \tag{S11}$$

Therefore, if

$$\begin{aligned} 1 - P_1/P_F^S &> P_1/P_k^S \\ \Leftrightarrow P_F^S P_k^S - P_1 P_k^S &> P_1 P_F^S \\ \Leftrightarrow P_1 &< P_F^S P_k^S \end{aligned} \tag{S12}$$

then the distribution is zero-inflated (i.e. the real P_0 is greater than the 'balanced' P_0). Recalling from Eq. S2 with $n = 1$ that

$$P_1 = (\tilde{s}P_k^S + \tilde{i}P_k^I)P_F^S, \tag{S13}$$

it follows that the condition for zero-inflation is equivalent to

$$\begin{aligned} \tilde{s}P_k^S + \tilde{i}P_k^I &< P_k^S \\ \Leftrightarrow P_k^I &< P_k^S \end{aligned} \tag{S14}$$

However, Eq. S8 can be rewritten

$$\begin{aligned} P_k^S &= P_k^I \frac{(1-w)\tilde{i}}{1-(1-w)\tilde{s}} \\ &= P_k^I \frac{(1-w)\tilde{i}}{(1-w)\tilde{i}+w} \\ &< P_k^I \end{aligned} \tag{S15}$$

and therefore since the pmf indicates that $P_k^S < P_k^I$ (Eq. S15) but zero-inflation requires that $\mathrm{P}_k^I < P_k^S$ (Eq. S14), it follows that the transmission distribution is always *zero-deflated*.

3 Moment generating function of the transmission distribution

Although we are mainly interested in the mean number of inoculations per feeding dispersal, we calculate the moment generating function as other moments, especially variance, may be of additional interest. The MGF satisfies:

$$
\begin{aligned}
M_x(t) &= \sum_{x=0}^{\infty} e^{tx} P_x \\
&= \tilde{s}P_F^S + \tilde{i}P_F^I + P_F^S(\tilde{s}P_k^S + \tilde{i}P_k^I) \sum_{x=1}^{\infty} P_k^{S\,x-1} e^{xt} \\
&= \tilde{s}P_F^S + \tilde{i}P_F^I + \frac{P_F^S}{P_k^S}(\tilde{s}P_k^S + \tilde{i}P_k^I) \sum_{x=1}^{\infty} (P_k^S e^t)^x \\
&= \tilde{s}P_F^S + \tilde{i}P_F^I + \frac{P_F^S}{P_k^S}(\tilde{s}P_k^S + \tilde{i}P_k^I)(\sum_{x=0}^{\infty} (P_k^S e^t)^x - 1) \qquad \text{(S16)} \\
&= \tilde{s}P_F^S + \tilde{i}P_F^I + \frac{P_F^S}{P_k^S}\frac{\tilde{s}P_k^S + \tilde{i}P_k^I}{1 - P_k^S e^t}(1 - (1 - P_k^S e^t)) \\
&= \tilde{s}P_F^S + \tilde{i}P_F^I + \frac{P_F^S}{P_k^S}\frac{\tilde{s}P_k^S + \tilde{i}P_k^I}{1 - P_k^S e^t} P_k^S e^t \\
&= \tilde{s}P_F^S + \tilde{i}P_F^I + P_F^S \frac{\tilde{s}P_k^S + \tilde{i}P_k^I}{1 - P_k^S e^t} e^t \qquad \text{(S17)}
\end{aligned}
$$

provided that $P_k^S e^t < 1$ for step S16. Differentiate with respect to t and evaluate at $t = 0$ to find the ith moment m_i. For instance, m_1, the mean number of inoculations per dispersal is:

$$
\begin{aligned}
m_1 = \frac{dM_x(t)}{dt}|_{t=0} &= P_F^S \frac{\tilde{s}P_k^S + \tilde{i}P_k^I}{1 - P_k^S e^t} e^t|_{t=0} + P_F^S \frac{\tilde{s}P_k^S + \tilde{i}P_k^I}{(1 - P_k^S e^t)^2} e^t P_k^S e^t|_{t=0} \\
&= P_F^S \frac{\tilde{s}P_k^S + \tilde{i}P_k^I}{1 - P_k^S e^t} e^t (1 + \frac{P_k^S e^t}{1 - P_k^S e^t})|_{t=0} \\
&= P_F^S \frac{\tilde{s}P_k^S + \tilde{i}P_k^I}{1 - P_k^S} (1 + \frac{P_k^S}{1 - P_k^S}) \\
&= P_F^S \frac{\tilde{s}P_k^S + \tilde{i}P_k^I}{(1 - P_k^S)^2} \\
&= \frac{\tilde{s}P_k^S + \tilde{i}P_k^I}{P_F^S} \\
&= \frac{\big(\tilde{s}(1-w)\tilde{i}(1-\epsilon w)\tilde{s} + \tilde{i}(1-\epsilon w)\tilde{s}(1-(1-w)\tilde{s})\big)}{(1-(1-\epsilon w))(1-(1-w)\tilde{s})} \frac{\big((1-(1-\epsilon w))(1-(1-w)\tilde{s})\big)}{(w\tilde{s} + \epsilon w\tilde{i})}
\end{aligned}
\tag{S18}
$$

where Eq. S18 follows from substituting in the expressions for P_F^S etc. from Eq.s S7-S10. Eq. S18 simplifies so that the mean number of inoculations, which henceforth is denoted by x, is given by

$$
x = \frac{\tilde{i}(1-\epsilon w)\tilde{s}}{\tilde{s}w + \tilde{i}\epsilon w}, \tag{S19}
$$

bearing in mind that $\tilde{s} = S/(S + \nu I)$ and $\tilde{i} = \nu I/(S + \nu I)$ throughout. Note that this appears as Eq. 1, main text. We can write out the mean in terms of the unbiased incidence of disease in the plant population ($i = I/(S + I)$ and $s = S/(S + I)$), and using the fact that $i = s - 1$, we can express the expression in terms of a single population variable i:

$$
\begin{aligned}
x &= \frac{(\nu I/(S+\nu I))(1-\epsilon w)(S/(S+\nu I))}{(S/(S+\nu I))w + (\nu I/(S+\nu I))\epsilon w} \\
&= \frac{vi(1-\epsilon w)}{w(1-i(1-\epsilon v))}\frac{(1-i)}{(1-i(1-v))}
\end{aligned} \tag{S20}
$$

4 Higher order correction to mean transmissions per dispersal

Eq. S20 (which was calculated from Eq.s S1-S2 together with Eq.s S3-S6) accurately represents mean transmission. However, a higher order correction can be made to take account of infecting the same plant more than once in a single dispersal. This correction will not have a significant impact under mean-field movement, as its effect is only to marginally adjust the probability of visiting susceptible plants. If l inoculations have occurred at a given stage of dispersal the corrected probability of visiting an S plant, denoted $\tilde{s}'$, is $\tilde{s}'(l) = (S-l)/(S+l+\nu I)$. Given the marginality of this effect except when field sizes are very small, for simplicity this correction is not implemented in this paper. However, we outline below how to take account of the correction.

At any stage in the dispersal the probabilities of feeding vs inoculating depend on the number of inoculations, l, so far made in the dispersal i.e. Eq.s S3-S6 become:

$$P_F^S(l) = 1.w + (1-w)\tilde{s}'(l)P_F^S(l) + (1-w)\tilde{i}P_F^I(l) \tag{S21}$$

$$P_F^I(l) = 1.\epsilon w + (1-\epsilon w)\tilde{s}'(l).0 + (1-\epsilon w)\tilde{i}P_F^I(l) \tag{S22}$$

$$P_k^S(l) = 0.w + (1-w)\tilde{s}'(l)P_k^S(l) + (1-w)\tilde{i}P_k^I(l) \tag{S23}$$

$$P_k^I(l) = 0.\epsilon w + (1-\epsilon w)\tilde{s}'(l).1 + (1-\epsilon w)\tilde{i}P_k^I(l) \tag{S24}$$

The *pmf* for the number of inoculations, n, per feeding dispersal of Eq.s S1-S2 becomes:

$$P_0 = \tilde{s}P_F^S(0) + \tilde{i}P_F^I(0) \tag{S25}$$

$$P_n = (\tilde{s}P_k^S(0) + \tilde{i}P_k^I(0))P_F^S(n)\prod_{m=1}^{n-1} P_k^S(m) \tag{S26}$$

Deriving a distribution for the number of transmissions per feeding dispersal

Given aphid is on either an S or an I plant, define two mutually exclusive subsequent events, A and B

		Probability	
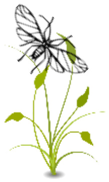	A, aphid feeds before inoculation is achieved	P_F^S	
	B, aphid inoculates a host plant before it accepts a plant for feeding	P_k^S	$(P_k^S = 1 - P_F^S)$
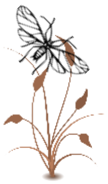	A, aphid feeds before inoculation is achieved	P_F^I	
	B, aphid inoculates a host plant before it accepts a plant for feeding	P_k^I	$(P_k^I = 1 - P_F^I)$

If feeding occurs first, then feeding dispersal has ended

If inoculation occurs first, then feeding dispersal continues, given aphid is now on an S plant (inoculation implies aphid is on an S plant)

In this way feeding dispersals can be represented in terms of the probabilities: P_F^S, P_F^I, P_k^S, P_k^I *which can be separately calculated (through conditioning on possible events).*

Figure S1: A schematic detailing notation and strategy for deriving the distribution for the number of transmission events per aphid feeding dispersal.
