# Supplementary material for: Pathogenic modification of plants enhances long‐distance dispersal of nonpersistently transmitted viruses to new hosts
Source: Ecology. 2019 May 21;100(7):e02725. doi: 10.1002/ecy.2725 (PMC6619343; doi:10.1002/ecy.2725)
Supplement: Supplementary file 2 [file ECY-100-na-s002.pdf]

Appendix S2, The processes governing the number of transmissions per feeding dispersal.

Mean number of transmissions per aphid feeding dispersal is

$$x(i) = \frac{\tilde{i}(1-\epsilon w)(1-\tilde{i})}{w(1-\tilde{i}(1-\epsilon))} \tag{S1}$$

consisting of a numerator representing the probability of visiting an infected plant, not feeding on it and then the probability of visiting a healthy plant (we refer to this as transmission probability). This is divided by a denominator representing the inverse of the expected number of visits in a feeding dispersal (i.e. longer feeding dispersals are associated with more opportunity for acquiring and inoculating the virus). Since Eq. S1 incorporates *VMPP*s we can analyze their effects by examining each of these terms.

In Fig.S1 the transmission probability is shown for varying viral incidence in the plant population with and without deterrence *VMPP* in Fig.S1A (i) and Fig.S1B (i) (with and without attraction also shown). The expected number of plants visited per dispersal with and without deterrence *VMPP* is shown in Fig.S1A (ii) and Fig.S1B (ii). Transmission distribution snapshots are shown in Fig.S1C for varying combinations of attraction, deterrence and incidence.

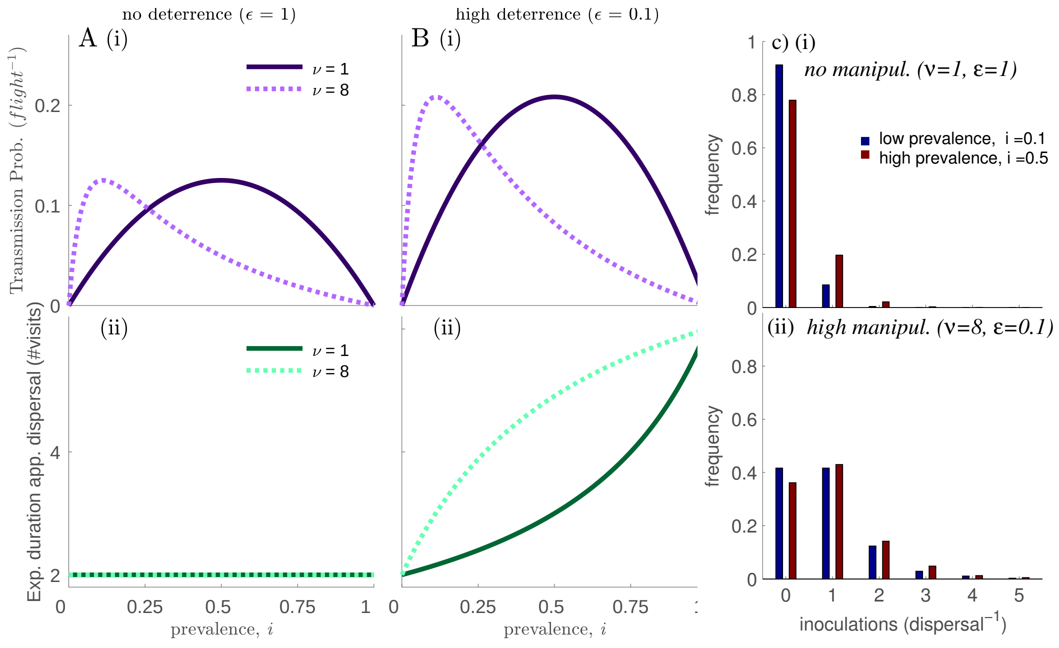

Figure S1: The probability of acquisition followed by inoculation is shown for varying viral incidence in plants for no deterrence, $\epsilon = 1$ (**A**, i), for high deterrence, $\epsilon = 0.1$ (**B**, i), with high attraction $\nu = 8$ represented by dashed curves and no attraction $\nu = 1$ by solid curves. The expected number of plants visited per dispersal is shown in $A\ (ii)$ for no deterrence and in $B\ (ii)$ for high deterrence. Snapshots of the transmission distribution are shown for no deterrence and no attraction in $C\ (i)$, for high deterrence and high attraction in $C\ (ii)$, with blue bars representing low incidence ($i = 0.1$) and red bars intermediate incidence ($i = 0.5$). A - C were generated using $w = 0.5$.
