# Supplementary material for: Pathogenic modification of plants enhances long‐distance dispersal of nonpersistently transmitted viruses to new hosts
Source: Ecology. 2019 May 21;100(7):e02725. doi: 10.1002/ecy.2725 (PMC6619343; doi:10.1002/ecy.2725)
Supplement: Supplementary file 3 [file ECY-100-na-s003.pdf]

Appendix S3, Extension of transmission distribution to incorporate per flight risk and imperfect acquisition/inoculation.

When there is a probability q, see Appendix S6: Fig.S1, of surviving each flight (and a probability $p = 1 - q$ of emigrating/dying each flight) it becomes convenient to define the mutually exclusive outcomes within dispersals as absorption vs inoculation. Absorption is defined as either feeding or loss of aphids through emigration/mortality (node *Loss* in Appendix S6: Fig.S1) and is denoted P_A^S and P_A^I, (note that the subscript A here is distinct from variable A in main text for total aphid population size). The probability of inoculation given departure from a plant of type S is $P_k^S = 1 - P_A^S$, and from a plant of type I is, $P_k^I = 1 - P_A^I$. In addition, the probabilities of acquisition and inoculation, P_{acq} and P_{inoc} respectively, are generally imperfect ($P_{acq} \leq 1$ and $P_{inoc} \leq 1$), i.e., when a virus-free aphid probes an infected plant virus acquisition occurs with probability P_{acq}. Similarly, when a virus-bearing aphid probes a healthy plant, inoculation occurs with probability P_{inoc}. The pmf, incorporating $P_{acq} \leq 1$, $P_{inoc} \leq 1$ and $q \leq 1$ is:

$$P_0 = p + q(\tilde{s}P_A^S + \tilde{i}P_A^I) \tag{S1}$$

$$P_n = q(\tilde{s}P_k^S + \tilde{i}P_k^I)(P_k^S)^{n-1}P_A^S \tag{S2}$$

with P_A^S, P_k^S, P_A^I, P_k^I all solutions of:

$$P_A^S = 1.w + q(1-w)\tilde{s}P_A^S + q(1-w)\tilde{i}P_A^I + (1-w)p \tag{S3}$$

$$P_A^I = 1.\epsilon w + P_{acq}P_{inoc}q(1-\epsilon w)\tilde{s}.0 + (1-P_{acq}P_{inoc})q(1-\epsilon w)\tilde{s}P_A^S + q(1-\epsilon w)\tilde{i}P_A^I + (1-\epsilon w)p \tag{S4}$$

$$P_k^S = 0.w + q(1-w)\tilde{s}P_k^S + q(1-w)\tilde{i}P_k^I + (1-w)p.0 \tag{S5}$$

$$P_k^I = 0.\epsilon w + P_{acq}P_{inoc}q(1-\epsilon w)\tilde{s}.1 + (1-P_{acq}P_{inoc})q(1-\epsilon w)\tilde{s}P_k^S + q(1-\epsilon w)\tilde{i}P_k^I + (1-\epsilon w)p.0 \tag{S6}$$

Combining Eq. S3 and S4 leads to:

$$P_A^I = \frac{(\epsilon w + (1-\epsilon w)p)(1-q(1-w)\tilde{s}) + (1-P_{acq}P_{inoc})q(1-\epsilon w)\tilde{s}(w+(1-w)p)}{(1-q(1-w)\tilde{s})(1-q(1-\epsilon w)\tilde{i}) - (1-P_{acq}P_{inoc})q(1-\epsilon w)\tilde{s}q(1-w)\tilde{i}} \tag{S7}$$

Combining Eq. S5 and S6 leads to:

$$P_k^I = \frac{(1-q(1-w)\tilde{s})P_{acq}P_{inoc}q(1-\epsilon w)\tilde{s}}{(1-q(1-w)\tilde{s})(1-q(1-\epsilon w)\tilde{i}) - (1-P_{acq}P_{inoc})q(1-\epsilon w)\tilde{s}q(1-w)\tilde{i}} \tag{S8}$$

Rearranging Eq. S3 and Eq. S5 leads to the relations:

$$P_A^S = \frac{w + (1-w)p + q(1-w)\tilde{i}P_A^I}{1-q(1-w)\tilde{s}} \tag{S9}$$

$$P_k^S = \frac{q(1-w)\tilde{i}P_k^I}{1-q(1-w)\tilde{s}} \tag{S10}$$

For simplicity, we do not give the closed form for P_k^S and P_A^S, but they are found by substituting

the expressions for P_k^I and P_A^I in Eq. S7 and S8 into Eq. S9 and S10.

The mean number of transmissions per dispersal from a healthy plant (i.e. calculating $\sum_{n=0}^{\infty} nP_n$ using Eq. S2) is

$$
\begin{aligned}
x =& q(\tilde{s}P_k^S + \tilde{i}P_k^I)P_A^S \sum_{n=1}^{\infty} n(P_k^S)^{n-1} && \text{(S11)}\\
=& q(\tilde{s}P_k^S + \tilde{i}P_k^I)P_A^S \frac{1}{(1-P_k^S)^2} && \text{(S12)}\\
=& q\frac{(\tilde{s}P_k^S + \tilde{i}P_k^I)}{P_A^S} && \text{(S13)}\\
=& \frac{P_k^S}{(1-w)P_A^S} && \text{(S14)}\\
=& \frac{q(1-w)\tilde{i}P_k^I}{(1-w)(w+(1-w)p+q(1-w)\tilde{i}P_A^I)} && \text{(S15)}
\end{aligned}
$$

where we have used the power series relation $\sum_{n=0}^{\infty} ny^{n-1} = 1/(1-y)^2$ to produce Eq. S12 from S11. We have used the relation $q(\tilde{s}P_k^S + \tilde{i}P_k^I) = P_k^S/(1-w)$, that is obtained from rearranging Eq. S5, to produce Eq. S14 from S13. In addition, we have used the relations from Eq. S9 and S10 to produce Eq. S15 from S14.

Finally, substituting the expressions for P_A^I and P_k^I from Eq. S7 and S8 into Eq. S15 leads to:

$$
x(i) = \frac{Pq(1-w)\tilde{i}}{(1-w)(Q(w+(1-w)p)+Rq(1-w)\tilde{i})} \qquad \text{(S16)}
$$

where P, Q and R are:

$$P = (1 - q(1-w)\tilde{s})P_{acq}P_{inoc}q(1-\epsilon w)\tilde{s} \tag{S17}$$

$$Q = (1 - q(1-w)\tilde{s} - q(1-\epsilon w)\tilde{i} + P_{acq}P_{inoc}q(1-\epsilon w)\tilde{s}q(1-w)\tilde{i} \tag{S18}$$

$$R = (\epsilon w + (1-\epsilon w)p)(1 - q(1-w)\tilde{s}) + (1 - P_{acq}P_{inoc})q(1-\epsilon w)\tilde{s}(w + (1-w)p) \tag{S19}$$

The expression for $x(i)$ in Eq. S16 can be further simplified through cancellation of the $P_{acq}P_{inoc}$ terms in the denominator. Further rearrrangement then leads to the expression:

$$x(i) = \frac{q^2 P_{acq}P_{inoc}\tilde{i}(1-\epsilon w)(1-\tilde{i})}{p + qw(1 - \tilde{i}(1-\epsilon))} \tag{S20}$$

For simplicity of presentation Eq. S20 with the values $q = 1$ and $P_{acq} = P_{inoc} = 1$ substituted in, appears as Eq. S19 in Appendix S1, Eq. S1 in Appendix S2 and as Eq. 1, main text. Note that though the equations shown in the main text are the result of substituting these fixed values of q, P_{acq} and P_{inoc} into Eq. S20 for simplicity, nevertheless our results are based on the general form as outlined in this appendix.
