# Supplementary material for: Pathogenic modification of plants enhances long‐distance dispersal of nonpersistently transmitted viruses to new hosts
Source: Ecology. 2019 May 21;100(7):e02725. doi: 10.1002/ecy.2725 (PMC6619343; doi:10.1002/ecy.2725)
Supplement: Supplementary file 4 [file ECY-100-na-s004.pdf]

Donnelly et al. Pathogenic modification of plants enhances long-distance dispersal of non-persistently transmitted viruses to new hosts. Ecology

Appendix S4, Deriving aphid dynamics from aphid feeding dispersals.

In Appendix S1 we derived a transmission distribution for aphid feeding dispersals. These equations can be accompanied by equations for aphid dynamics to form pathosystem dynamics.

Per plant aphid abundance for susceptible and infected plants, denoted A_S and A_I respectively, satisfy Eq.s $6-7$ main text. For convenience these equations are reproduced here:

$$\frac{dA_S}{dt} = aA_S(1 - A_S/\kappa) - bA_S - \theta A_S(1 - F_S) + \theta A_I F_S i/(1-i) \tag{S1}$$

$$\frac{dA_I}{dt} = aA_I(1 - A_I/\kappa) - bA_I - \theta A_I(1 - F_I) + \theta A_S F_I (1-i)/i \tag{S2}$$

where parameters are as per main text and Table 2. In Eq.s S1-S2 F_S denotes the probability of settling on an S plant (and analogously F_I for settling on a type I plant). Conditioning on the possible dispersal events leads to:

$$F_S = q(\tilde{s}(1-w)F_S + \tilde{i}(1-\epsilon w)F_S + \tilde{s}w) \tag{S3}$$

$$F_I = q(\tilde{s}(1-w)F_I + \tilde{i}(1-\epsilon w)F_I + \tilde{i}\epsilon w) \tag{S4}$$

In Eq.s S3-S4 the first term on the right hand side conditions on the next event being a visit to an S, or then an I (second term) plant, without acceptance. The second term conditions on visits in the same way but with acceptance.

Solving Eq S3 leads to:

$$F_S = \frac{q\tilde{s}w}{1 - q(\tilde{s}(1-w) + \tilde{i}(1-\epsilon w))} = \frac{qSw}{q(Sw + \nu\epsilon wI) + p} \tag{S5}$$

Solving Eq S4 leads to:

$$F_I = \frac{q\tilde{i}\epsilon w}{1 - q(\tilde{s}(1-w) + \tilde{i}(1-\epsilon w))} = \frac{q\nu\epsilon wI}{q(Sw + \nu\epsilon wI) + p} \tag{S6}$$

This allows Eqs. S1-S2 to be solved for a given value of i, with i itself satisfying the following equation:

$$\frac{di}{dt} = \frac{\theta((1-i)HA_S + iHA_I)}{H} x(i) - \Gamma i, \tag{S7}$$

and with A_S and A_I the solutions of Eq.s S1-S2.
