# Supplementary material for: Pathogenic modification of plants enhances long‐distance dispersal of nonpersistently transmitted viruses to new hosts
Source: Ecology. 2019 May 21;100(7):e02725. doi: 10.1002/ecy.2725 (PMC6619343; doi:10.1002/ecy.2725)
Supplement: Supplementary file 5 [file ECY-100-na-s005.pdf]

# Appendix S5, Description of stochastic simulations.

*Appendix S1* introduced distributions for the number of inoculations per feeding dispersal which, together with expressions or equations for aphid dynamics, form a deterministic model of disease progress. An alternative method for studying disease progress is through stochastic simulation, which incorporates the randomness inherent in demographic change. To implement a stochastic simulation we define population variables (representing interacting organisms e.g. healthy plants, aphids, virus-infected plants) together with life-history events which alter these variable levels, and the rates at which events occur. By randomly simulating 1) the time until the next event, according to the total of all rates, and 2) deciding which event has occurred, by simulating in proportion to a particular event's relative rate, i.e. using a Gillespie algorithm (Gillespie , 1977), we stochastically simulate aphid dynamics and the spread of viral infection in the host plant population (Table S1).

For convenience, we describe this for the case of mean-field aphid movement in Table S1. However, we extended the simulation procedure for wingless aphids by making the simulation spatially explicit, i.e. defining an array of plants to represent a field. Each plant is either healthy or virus-infected and has a density of winged and wingless aphids. Wingless aphid movement is constrained to plant to neighboring plant, winged aphids move to any plant in the population. In simulations with concurrent winged and wingless aphids each plant has distinct populations of winged and wingless aphids. However, each aphid on an individual plant has the same per capita reproduction rate, whether winged or wingless corresponding to $a(1 - A_{i,j}/\kappa)$ where $A_{i,j}$ is the total number of winged and wingless aphids on the plant in row $i$, column $j$. Offspring born to aphids on plant $i, j$ are winged according to the relationship $Prob(winged) = A_{i,j}^6/(A_{i,j}^6 + (0.5\kappa)^6)$ i.e., the probability that an offspring develops to be

winged is a function of the magnitude of $A_{i,j}$ to reflect the effect of local crowding on development into a winged form. In the simulations a random uniform number on the interval $[0,1]$, $r$, is drawn each time an aphid is born and is compared to $Prob(winged)(A_{i,j})$ to determine if the offspring is winged or wingless. If $r < Prob(winged)$ the offspring is winged, otherwise it is wingless. The stochastic simulation code was run on MATLAB 2018a and the code is available on GitHub (Donnelly, 2018).

| **A** *(i) Population variables* | | *Units* |
|---|---|---|
| $I$ | # Infected plants | *per field* |
| $S$ | # Healthy plants | *per field* |
| $A$ | # Winged aphids | *per field/type j plant* |
| $A_{ij}$ | # Winged aphids on individual plant | *per plant at position $i,j$* |
| *(ii) Parameters* | | |
| $p$ | Aphid dies/emigrates when switching plants | *prob. per journey* |
| $\nu$ | Infected plant bias (Pl. attractiveness VMPP) | *multiplicative factor* |
| $w$ | Acceptance rate (healthy plant) | *prob. after probing* |
| $\epsilon$w | Acceptance rate (infected plant) | *prob. after probing* |
| *(iii) Rates (total)* | | |
| ΓI | Cease plant infectiousness | *per day* |
| $bA$ | Aphid dies | *per day* |
| $\theta A$ | Aphid dispersal | *per day* |
| $aA_{i,j}(1 - A_{i,j}/K)$ | Aphid reproduction on plant $i,j$ | *per day* |

| **B.** *Algorithm* | *Corresponding change to variable* |
|---|---|
| while t<T | |
| sample $\tau$ from exponential with mean=total rates | $t = t + \tau$ |
| choose event proportional to relative rates using uniform rv | |
| if event is: infected plant death/recovery | *I=I-1* |
| aphid death | *A=A-1* |
| aphid reproduction | *A=A+1* |
| aphid dispersal: enter dispersal sub-routine | *A=A-1* |
| while aphid has not yet accepted plant | |
| die/emigrate if uniform rv $< p$; break | |
| go to I if uniform rv $< \frac{\nu I}{S+\nu I}$ | *Dispersal outcomes* |
| accept plant if uniform rv $< \epsilon w$; break | *A=A+1* |
| else acquire virus | $A^{+}$ (i.e. aphid has virus) |
| go to S if uniform rv $\geq \frac{\nu I}{S+\nu I}$ | |
| if aphid has virus (i. e., $A^{+}$), infect plant | *I=I+1* |
| accept plant if uniform rv $< w$; break | *A=A+1* |
| end while | |
| end while | |

Table S1: Stochastic simulation of the spread of NPT virus infection through a population of host plants and vectored by aphids. **A** Definition of notation, parameters and rates used. **B** The structure of the Gillespie algorithm used as well as the sub-routine for plant selection in which new plant infections occur as a by-product of feeding dispersals of winged aphids.

# References

Donnelly, R. 2018 Supporting computer code for, Pathogenic modification of plants enhances long-distance dispersal of non-persistently transmitted viruses to new hosts. GitHub repository, DOI: https://zenodo.org/badge/latestdoi/147835191

Gillespie, D.T. 1977 Exact stochastic simulation of coupled chemical reactions. The Journal of Physical Chemistry, 81(25), pp.2340-2361
