# Supplementary material for: Pathogenic modification of plants enhances long‐distance dispersal of nonpersistently transmitted viruses to new hosts
Source: Ecology. 2019 May 21;100(7):e02725. doi: 10.1002/ecy.2725 (PMC6619343; doi:10.1002/ecy.2725)
Supplement: Supplementary file 6 [file ECY-100-na-s006.pdf]

Appendix S6, Extension of methods to feeding dispersals of wingless aphids.

1 Number of NPT transmissions per wingless feeding dispersal

For simplicity, we do not mathematically model transmission due to wingless aphids in the main text. However, the methods from Appendix S1 can be extended to wingless aphids. In this section we sketch out the calculations. The formulation can be used to provide an expression for force of infection due to vectoring by wingless aphids, that may prove useful in theoretical studies as well as statistical fitting.

Analogously to Appendix S1 we consider two alternative outcomes of a feeding dispersal: a) inoculation b) mortality/emigration/feeding (i.e. absorption). The Markov chain, which is analogous to Fig. 1B main text, is displayed in Fig. S1. Note that the term $P_{X|Y}$ represents the probability that a neighbor of a plant of type Y is of type X. For example, $P_{S|I}$, is the probability that a neighboring plant to an infected plant is healthy. Proceeding as per Appendix S1, the pmf for the number of inoculations per feeding dispersal starting from an S plant is:

$$P_0 = p + q(\tilde{P}_{S|S}P_A^S + \tilde{P}_{I|S}P_A^I) \tag{S1}$$

$$P_n = q(\tilde{P}_{S|S}P_k^S + \tilde{P}_{I|S}P_k^I)(P_k^S)^{n-1}P_A^S \tag{S2}$$

where $\tilde{P}_{S|S}$ and $\tilde{P}_{S|I}$ represents the probability of the vector moving to a susceptible plant and $\tilde{P}_{I|S}$ and $\tilde{P}_{I|I}$ on an infected plant, e.g. $\tilde{P}_{S|S} = P_{S|S}/(P_{S|S} + \nu P_{I|S})$ and $\tilde{P}_{I|S} = \nu P_{I|S}/(P_{S|S} + \nu P_{I|S})$, since the landing probabilities incorporate a bias, $\nu > 1$, due to *VMPP*. The probabilities P_F^S, P_F^I, P_k^S and P_k^I are the solutions of the relations

$$P_A^S = 1.w + q(1-w)\tilde{P}_{S|S}P_A^S + q(1-w)\tilde{P}_{I|S}P_A^I + (1-w)p \tag{S3}$$

$$P_A^I = 1.\epsilon w + q(1-\epsilon w)\tilde{P}_{S|I}.0 + q(1-\epsilon w)\tilde{P}_{I|I}P_A^I + (1-\epsilon w)p \tag{S4}$$

$$P_k^S = 0.w + q(1-w)\tilde{P}_{S|S}P_k^S + q(1-w)\tilde{P}_{I|S}P_k^I + (1-w)p0 \tag{S5}$$

$$P_k^I = 0.\epsilon w + q(1-\epsilon w)\tilde{P}_{S|I}.1 + q(1-\epsilon w)\tilde{P}_{I|I}P_k^I + (1-\epsilon w)p0 \tag{S6}$$

17 Solving these simultaneous equations results in the following expressions

$$P_A^S = \frac{(w+(1-w)p)(1-q(1-\epsilon w)\tilde{P}_{I|I}) + q(1-w)\tilde{P}_{I|S}(\epsilon w + (1-\epsilon w)p)}{(1-q(1-\epsilon w)\tilde{P}_{I|I})(1-q(1-w)\tilde{P}_{S|S})} \tag{S7}$$

$$P_k^S = \frac{q(1-w)\tilde{P}_{I|S}(1-\epsilon w)q\tilde{P}_{S|I}}{(1-q(1-\epsilon w)\tilde{P}_{I|I})(1-q(1-w)\tilde{P}_{S|S})} \tag{S8}$$

$$P_A^I = \frac{\epsilon w + (1-\epsilon w)p}{1-q(1-\epsilon w)\tilde{P}_{I|I}} \tag{S9}$$

$$P_k^I = \frac{q(1-\epsilon w)\tilde{P}_{S|I}}{1-q(1-\epsilon w)\tilde{P}_{I|I}} \tag{S10}$$

18 Eq.s S7-S10 together with S1-S2 fully specify the distribution for the number of inoculations
19 per wingless feeding dispersal from a healthy plant. Mean transmissions per dispersal from *a*
20 *healthy plant* is then:

$$x_S = q(\tilde{P}_{S|S}P_k^S + \tilde{P}_{I|S}P_k^I)P_A^S \sum_{n=1}^{\infty} n(P_k^S)^{n-1} \tag{S11}$$

$$= q(\tilde{P}_{S|S}P_k^S + \tilde{P}_{I|S}P_k^I)P_A^S \frac{1}{(1-P_k^S)^2} \tag{S12}$$

$$= q\frac{(\tilde{P}_{S|S}P_k^S + \tilde{P}_{I|S}P_k^I)}{P_A^S} \tag{S13}$$

Mean transmissions per dispersal from *an infected plant* (i.e. exactly the same as for from a healthy plant but with initial movement from I rather than S in Eq. S2) is

$$x_I = q(\tilde{P}_{S|I}P_k^S + \tilde{P}_{I|I}P_k^I)P_A^S \sum_{n=1}^{\infty} n(P_k^S)^{n-1} \tag{S14}$$

$$= q(\tilde{P}_{S|I}P_k^S + \tilde{P}_{I|I}P_k^I)P_A^S \frac{1}{(1-P_k^S)^2} \tag{S15}$$

$$= q\frac{(\tilde{P}_{S|I}P_k^S + \tilde{P}_{I|I}P_k^I)}{P_A^S} \tag{S16}$$

2 Wingless aphid dynamics

In *section 1* of this appendix we derived transmission distributions for wingless aphids. These equations can be accompanied by equations for wingless aphid dynamics to form pathosystem dynamics when aphids are wingless. Per plant wingless aphid abundance for susceptible and infected plants, denoted A_S and A_I respectively, satisfy the same equations as for winged aphids i.e. Eq.s $5-6$ main text. For convenience these equations are reproduced here:

$$\frac{dA_S}{dt} = aA_S(1 - A_S/\kappa) - bA_S - \theta A_S(1 - F_S^S) + \theta A_I F_S^I i/(1-i) \tag{S17}$$

$$\frac{dA_I}{dt} = aA_I(1 - A_I/\kappa) - bA_I - \theta A_I(1 - F_I^I) + \theta A_S F_I^S(1-i)/i \tag{S18}$$

where parameters are as per main text and Table 2. In Eq.s S17-S18 F_S^S denotes the probability of settling on an S plant given departure from S plants (and analogously F_S^I for departing an I plant, and similarly F_I^S and F_I^I for settling on a type I plant given departure from S and I type plants respectively). Conditioning on the possible dispersal events leads to:

$$\begin{pmatrix} F_I^S \\ F_I^I \end{pmatrix} = q \begin{pmatrix} \tilde{P}_{S|S}(1-w) & \tilde{P}_{I|S}(1-\epsilon w) \\ \tilde{P}_{S|I}(1-w) & \tilde{P}_{I|I}(1-\epsilon w) \end{pmatrix} \begin{pmatrix} F_I^S \\ F_I^I \end{pmatrix} + q \begin{pmatrix} \tilde{P}_{S|S}w & \tilde{P}_{I|S}\epsilon w \\ \tilde{P}_{S|I}w & \tilde{P}_{I|I}\epsilon w \end{pmatrix} \begin{pmatrix} 0 \\ 1 \end{pmatrix} \tag{S19}$$

In Eq. S19 the first matrix on the right hand side conditions on the next event being a visit to an S (column 1), or then an I (column 2) plant, without acceptance. The second matrix conditions on visits in the same way but with acceptance. Note that Eq. S19 determines the probabilities of settling on an infected plant $(F_I^S \quad F_I^I)^T$ but if the final vector is instead $(1 \quad 0)^T$ it becomes an equation for the probabilities of settling on healthy plants, i.e. dependent variables are $(F_S^S \quad F_S^I)^T$. This allows Eqs. S17-S18 to be solved for a given value of i and $P_{I|I}$, with i itself satisfying the following equation when aphids are wingless:

$$\frac{di}{dt} = \theta \left(\frac{(1-i)HA_S}{H} x_S(i) + \frac{iHA_I}{H} x_I(i) \right) - \Gamma i, \tag{S20}$$

and with A_S and A_I the solutions of Eqs. S17-S18.

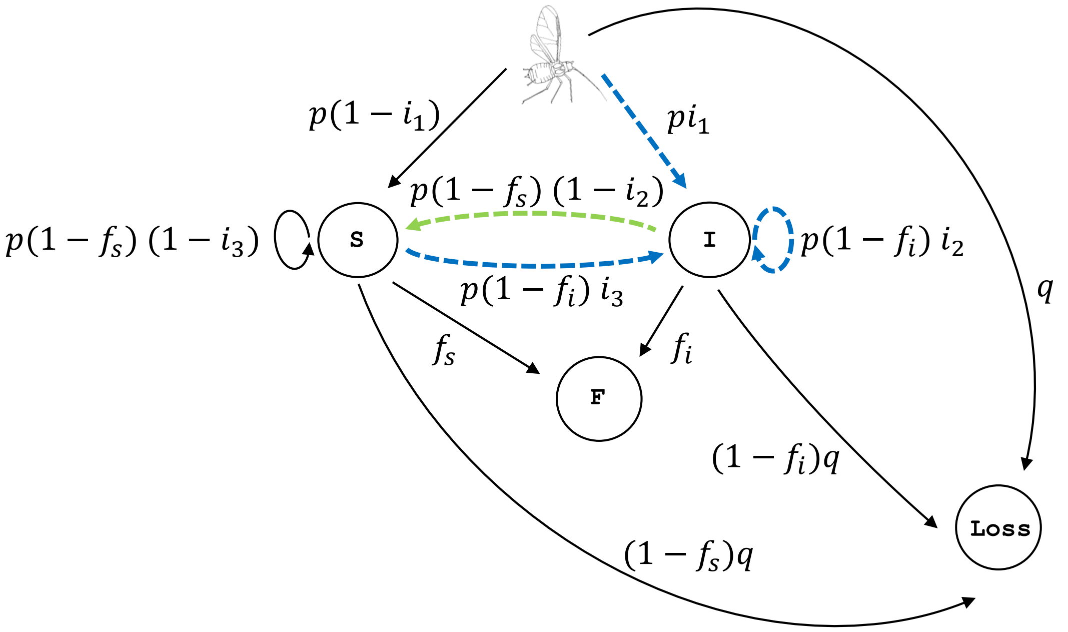

Transition probabilities

	Winged 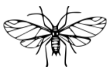	Wingless (from S plant) 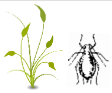	Wingless (from I plant) 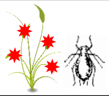
$i_1=$	$\nu I/(S+\nu I)$	$\nu P_{I\vert S}/(P_{S\vert S}+\nu P_{I\vert S})$	$\nu P_{I\vert I}/(P_{S\vert I}+\nu P_{I\vert I})$
$i_2=$	$\nu I/(S+\nu I)$	$\nu P_{I\vert I}/(P_{S\vert I}+\nu P_{I\vert I})$	$\nu P_{I\vert S}/(P_{S\vert S}+\nu P_{I\vert S})$
$i_3=$	$\nu I/(S+\nu I)$	$\nu P_{I\vert S}/(P_{S\vert S}+\nu P_{I\vert S})$	$\nu P_{I\vert S}/(P_{S\vert S}+\nu P_{I\vert S})$
$f_s=$	w	w	w
$f_i=$	ϵw	ϵw	ϵw

Figure S1: Markov-chain representing general winged or wingless aphid feeding dispersals. In the case of wingless the plant from which dispersal began becomes important (hence two columns in table). See edges between nodes for transition probabilities. The blue and green arrows indicate acquisition and inoculation of NPT virus respectively. The loss node corresponds to the state of aphid emigration or death associated with journeys between plants.
