# Supplementary material for: Pathogenic modification of plants enhances long‐distance dispersal of nonpersistently transmitted viruses to new hosts
Source: Ecology. 2019 May 21;100(7):e02725. doi: 10.1002/ecy.2725 (PMC6619343; doi:10.1002/ecy.2725)
Supplement: Supplementary file 7 [file ECY-100-na-s007.pdf]

# Appendix S7, Comparison of epidemic trajectories for VMPP combinations when aphids are exposed to movement risks.

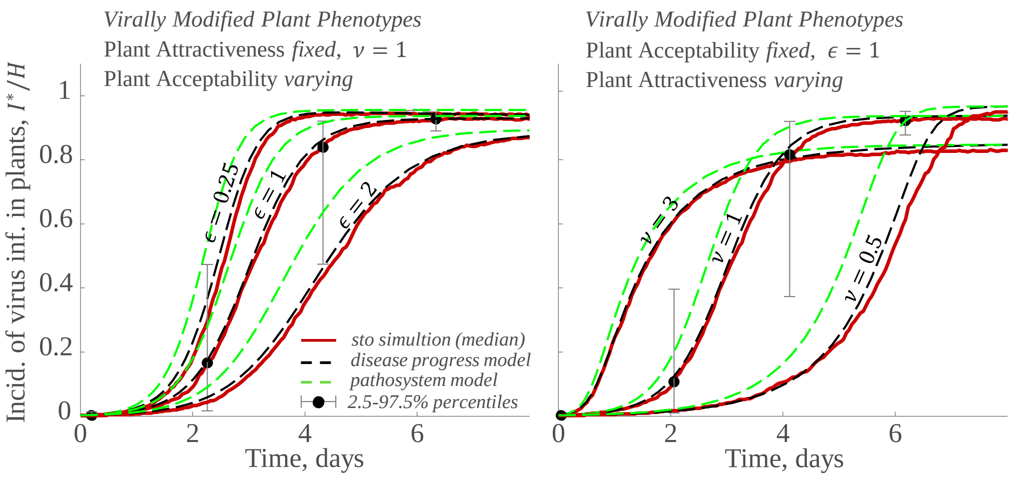


Figure S1

Figure S1: Comparison of epidemic trajectories for different VMPP combinations when aphids are exposed to risks associated with movement (i.e., $p > 0$). **A** Trajectories are shown for contrasting values of plant acceptability VMPP, $\epsilon$. Aphids have no preference among plants (i.e., no effect of virus infection on plant attractiveness, $\nu = 1$). **B** Trajectories are shown for contrasting values of plant attractiveness VMPP, $\nu$. Aphids accept plants at the same rate (i.e., no effect of infection on plant acceptability, $\epsilon = 1$). Red curves represent median of event-based simulations. In A and B black dashed curves represent solutions of the mathematical model (i.e., Eq.2 was solved using MATLAB version 2018a ode45 solver) taking the median aphid population size from the simulations as $A(t)$. Green dashed curves represent the mathematical model from Eq. 2 together with the solutions of eq.'s 6-7 for aphid density. Parameters were as per caption of Fig. 2, main text, but in addition aphid dynamics were included by incorporating aphid reproduction and natural mortality at the following rates per day: *a=2, b=1/10*. Additionally: *κ=10 and p=0.2*. Medians were calculated from 100 simulations over $20 \times 20$ plants.
