# Supplementary material for: Pathogenic modification of plants enhances long‐distance dispersal of nonpersistently transmitted viruses to new hosts
Source: Ecology. 2019 May 21;100(7):e02725. doi: 10.1002/ecy.2725 (PMC6619343; doi:10.1002/ecy.2725)
Supplement: Supplementary file 8 [file ECY-100-na-s008.pdf]

Appendix S8, The impact of NPT virus epidemiology on the concurrent transient dynamics of winged and wingless aphids.

Concurrent winged and wingless dynamics

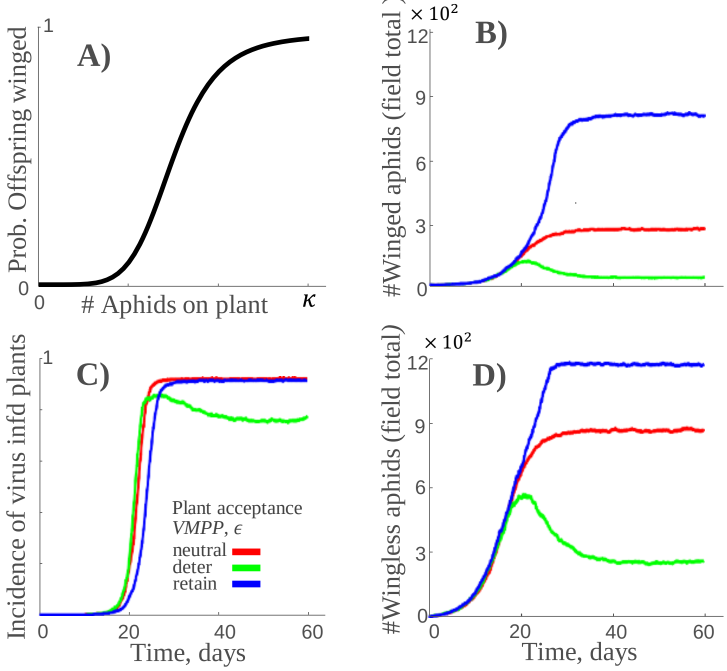

Figure S1: The impact of NPT virus epidemiology on the concurrent transient dynamics of winged and wingless aphids. **A** High aphid density on individual plants causes offspring to develop as the winged form. **B** & **D** Population dynamics of winged (B) and wingless (D) aphids, as the epidemic progresses. **C** Incidence of virus infected plants. Parameters for $A - D$ were as per caption of Fig. 4, main text. The probability that individual offspring develop as winged on plant i, j was $A_{i,j}^6/(A_{i,j}^6 + (0.5\kappa)^6)$, where form was chosen so probability offspring is winged is 0.5 when $A_{i,j} = \kappa/2$, and in addition, is higher than 0.95 when $A_{i,j} = \kappa$. All figures were generated using median of 4000 replicate simulations commencing with simultaneous invasion of virus and single settled winged aphid into a field of 20×20 susceptible host plants.
