# Supplementary material for: Pathogenic modification of plants enhances long‐distance dispersal of nonpersistently transmitted viruses to new hosts
Source: Ecology. 2019 May 21;100(7):e02725. doi: 10.1002/ecy.2725 (PMC6619343; doi:10.1002/ecy.2725)
Supplement: Supplementary file 9 [file ECY-100-na-s009.pdf]

Appendix S9, Markov chain model for virus transmission in a population of susceptible, infected and virus-resistant plants.

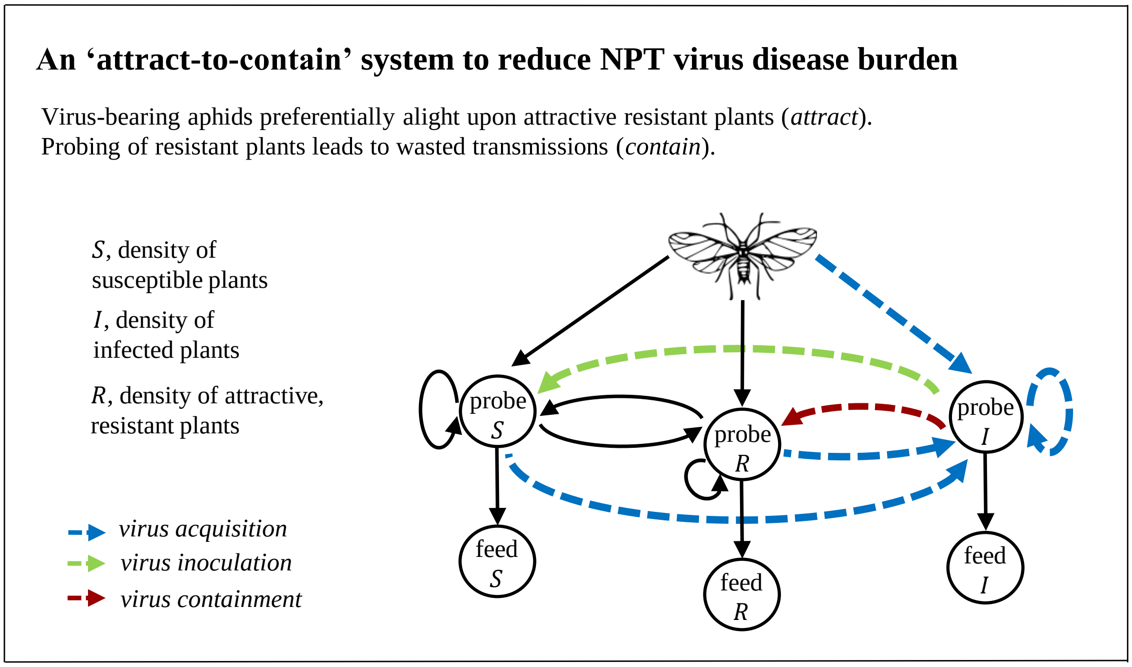

Figure S1: Markov chain model for virus transmission during individual feeding dispersals of aphid vectors in a population of infected (I) and susceptible (S) plants and resistant (R) plants. In addition to virus acquisition (blue arrow), and virus inoculation (green arrow), the presence of resistant plants allows for the possibility of virus disinfection of virus-bearing aphids, when they probe resistant plants (red arrow).
